# Microplastics in the aquatic and terrestrial environment: sources (with a specific focus on personal care products), fate and effects

**DOI:** 10.1186/s12302-015-0069-y

**Published:** 2016-01-06

**Authors:** Karen Duis, Anja Coors

**Affiliations:** ECT Oekotoxikologie GmbH, Böttgerstr. 2-14, 65439 Flörsheim/Main, Germany

**Keywords:** Plastic debris, Environmental concern, Persistence, Personal care products, Cosmetic products, Microplastic

## Abstract

Due to the widespread use and durability of synthetic polymers, plastic debris occurs in the environment worldwide. In the present work, information on sources and fate of microplastic particles in the aquatic and terrestrial environment, and on their uptake and effects, mainly in aquatic organisms, is reviewed. Microplastics in the environment originate from a variety of sources. Quantitative information on the relevance of these sources is generally lacking, but first estimates indicate that abrasion and fragmentation of larger plastic items and materials containing synthetic polymers are likely to be most relevant. Microplastics are ingested and, mostly, excreted rapidly by numerous aquatic organisms. So far, there is no clear evidence of bioaccumulation or biomagnification. In laboratory studies, the ingestion of large amounts of microplastics mainly led to a lower food uptake and, consequently, reduced energy reserves and effects on other physiological functions. Based on the evaluated data, the lowest microplastic concentrations affecting marine organisms exposed via water are much higher than levels measured in marine water. In lugworms exposed via sediment, effects were observed at microplastic levels that were higher than those in subtidal sediments but in the same range as maximum levels in beach sediments. Hydrophobic contaminants are enriched on microplastics, but the available experimental results and modelling approaches indicate that the transfer of sorbed pollutants by microplastics is not likely to contribute significantly to bioaccumulation of these pollutants. Prior to being able to comprehensively assess possible environmental risks caused by microplastics a number of knowledge gaps need to be filled. However, in view of the persistence of microplastics in the environment, the high concentrations measured at some environmental sites and the prospective of strongly increasing concentrations, the release of plastics into the environment should be reduced in a broad and global effort regardless of a proof of an environmental risk.

**Electronic supplementary material:**

The online version of this article (doi:10.1186/s12302-015-0069-y) contains supplementary material, which is available to authorised users.

## Introduction

World production of plastics (i.e. synthetic organic polymers) has strongly expanded during the last decades, from 1.7 million t in 1950 to 299 million t in 2013. While the amount of plastics produced in Europe has been relatively constant in the last 10 years, world plastic production continues to increase [1, 2]. In view of the large production volumes and the durability of plastics, it is not surprising that plastics are found in the environment. Initially, scientific and public attention focused on larger plastic debris. However, the occurrence of small plastic particles in the marine environment was already described in the early 1970s [3, 4]. During the last few years, microplastics in the environment have received increasing attention and are now an emerging area of research [5–8].

Most commonly, microplastics have been defined as synthetic organic polymer particles with a size (or, more specifically, largest dimension) <5 mm [6, 8–11]. The majority of definitions do not include a lower size limit. In view of the definition of nanoscale (1–100 nm [12]), the term microplastics is used in this review for solid synthetic organic polymer particles with a size between 100 nm and 5 mm. In studies on the occurrence in the environment, the upper size limit of the sampled plastics is not always indicated. In such cases, the term microplastics is used, if it can be assumed that the sampled plastic items are in the size range mentioned above. In cases where sampling included microplastics, but the upper size limit of the sampled plastics is somewhat above 5 mm (e.g. 10 mm) the term ‘small plastic items’ is employed. Plastic items larger than 5 mm are designated as macroplastics.

In the present work, currently available information on sources, fate and occurrence of microplastics in the aquatic and terrestrial environment, on their uptake by aquatic and terrestrial organisms and possible effects on these organisms is critically evaluated.

Recently, considerable public attention has focused on microplastics particles from personal care products, which was mainly triggered by reports in news media. Therefore, a specific focus is placed on the contribution of microplastics from personal care products (defined and regulated as cosmetic products in the EU [13]) to the overall pollution of the environment with microplastics.

An effort was made to report, as far as possible, numerical concentrations of microplastics for studies on occurrence, uptake and effects. Plastic products may contain a number of additives including plasticisers, stabilisers, flame retardants, pigments and antimicrobials [14–16]. Potential effects of these additives, which have been discussed elsewhere (e.g. [17]), are not addressed in this review.

## Methods to sample, process and analyse microplastics in the environment

A number of methods to sample, isolate, characterise, identify and quantify microplastics have been developed for water and sediment. In the following sections, the most relevant methods are briefly presented. In view of the comprehensive review of Hidalgo-Ruz et al. [11] and available guidance on monitoring of marine litter including microplastics [6], emphasis is placed on recent developments.

### Sampling

While in some cases bulk water samples were taken (e.g. [18]), volume-reduced sampling methods have generally been employed to sample microplastics from water: neuston nets for the sea surface layer, and zooplankton nets for sub-surface water [6, 11, 19, 20]. Mesh width of the sampling nets was most commonly 300–390 µm [11]. The sampling method has a strong influence on the study results, especially concerning the smallest microplastics, which require a sufficiently small mesh width or bulk sampling, depending on their size [21].

Samples from subtidal sediments are taken with sediment sampling equipment, e.g. grab samplers [6, 11, 22]. For coastal sediments, direct sampling of visually identified microplastics (e.g. by hand or using tweezers) has been used. This method can be employed to sample larger microplastics such as plastic resin pellets (typical diameter: 3.5 mm [23]) from the surface of sandy beaches. However, it is not suitable for sampling microplastics that do not have a characteristic shape, are smaller than plastic resin pellets or are mixed with other debris [6, 11]. In these cases, bulk sampling is required to avoid an underrepresentation of small microplastics. As the distribution of microplastics on beaches is often heterogeneous, attention needs to be paid to sample size, replication and the location of the sampling sites on the beach [8].

### Sample processing

To recover microplastics from bulk or volume-reduced water samples, a sieving or filtration step is commonly used. Alternatively, microplastics have been collected from the surface of the water sample using tweezers. Yet, there is a high likelihood of bias when visually collecting microplastics [11, 24]: the lowest particle size in studies, in which sieves were used, was smaller than in studies, in which particles were visually collected from the samples [11].

Sieving and, especially, density separation are used to extract microplastics from bulk sediment samples. Plastic particles usually have a much lower specific weight (Table 1) than sediments (typically 2.65 g/cm^3^ [11]). When sediment samples are mixed with salt solution of an appropriate density, sediment settles at the bottom, while microplastics can be collected from the surface [6, 11, 25]. Examples of such salt solutions are concentrated sodium chloride (density: 1.2 g/cm^3^), sodium polytungstate (1.4 g/cm^3^), sodium iodide (1.6–1.8 g/cm^3^) and zinc chloride (1.5–1.7 g/cm^3^) [6, 26–29]. To separate all plastic resin types from the sediment, salt solutions with a density ≥1.45 g/cm^3^ have been recommended [26, 29]. Several devices are available for density separation [26–28]. The resultant salt solution is subjected to sieving or filtration.

**Table 1:** Densities of plastic materials that are often found in the aquatic environment

Plastic class	Abbreviation	Density (g/cm^3^)^a,b^
Expanded polystyrene (styrofoam)	EPS	0.01–0.04
Low-density polyethylene	LDPE	0.89–0.93
High-density polyethylene	HDPE	0.94–0.98
Polypropylene	PP	0.83–0.92
Polyethylene terephthalate	PET	0.96–1.45
Polyamide (nylon)	PA	1.02–1.16
Polystyrene	PS	1.04–1.1
Polymethyl methacrylate (acrylic)	PMMA	1.09–1.20
Polyvinylchloride	PVC	1.16–1.58
Polycarbonate	PC	1.20–1.22
Polyurethane	PU	1.2
Alkyd	–	1.24–2.10
Polyester	PES	1.24–2.3
Polytetrafluoroethylene	PTFE	2.1–2.3

^a^Note that densities of plastic items can be modified by additives and environmental processes such as weathering and fouling

^b^Based on [11, 16, 25, 109]

To separate microplastics from other material recovered by the abovementioned sieving, filtration or density separation procedures, visual sorting has in most cases been used. However, visual differentiation of microplastic particles from other debris or grains of sand is difficult and only suitable for microplastics larger than approx. 1 mm [6, 11, 22, 30]. Transparent or white particles require careful differentiation methods, e.g. examination under high magnification using a fluorescence microscope to confirm the absence of cellular structures [11, 30].

Recently, methods for chemical and enzymatic clean-up have been developed. Treatment of samples with chemicals (e.g. 30–35 % H_2_O_2_, 20 % HCl, 30 % NaOH), partly combined with increased temperatures, has been used to remove organic material and calcified structures. However, chemical treatment may lead to a partial or complete degradation of microplastics [24, 26, 28, 31–33]. For this reason, digestion with enzymes (proteinase, cellulase, lipase and chitinase) has been recommended to separate microplastics from organic material [31, 32]. Chemical (e.g. 69 % HNO_3_, 10 % KOH, 30 % H_2_O_2_) and enzymatic (proteinase) methods have also been used to extract microplastics from tissues of aquatic organisms and from the digestive tract content of fish [32–39].

### Identification and quantification

Characteristics such as density and colour have been used to identify the polymer type of microplastics. However, these characteristics can be affected by weathering. As mentioned above, non-plastic microparticles and microfibers may be wrongly classified as microplastics [11, 30, 40]. When analysing microparticles, which had been visually classified as microplastics, with Fourier transform infrared spectroscopy (FT-IR), up to 70 % of the particles were not confirmed to be plastics [11]. Using scanning electron microscopy combined with energy dispersive X-ray spectroscopy (SEM–EDS), Eriksen al. [41] showed that numerous particles, which had been visually identified as microplastics, were aluminium silicates from coal ash and coal fly ash. Consequently, further analyses are required (1) to unequivocally identify microplastics and (2) to obtain information on their resin composition [6, 8, 11, 24, 30, 40, 41]. The use of infrared (IR) and Raman spectroscopy has been highly recommended for this purpose [11, 30]. In EC [6], it is suggested that all particles with a size between 20 and 100 µm and 5–10 % of the particles with a size between 100 µm and 5 mm should be further analysed by IR or Raman spectroscopy. Infrared spectrophotometry and FT-IR are probably the most commonly used method to identify the chemical composition of microplastics. If coupled with microscopy they can be used to identify microplastics with a size >20 µm [6, 11, 30, 31]. Raman spectroscopy combined with microscopy has a higher resolution (approx. 1–2 µm [26, 30, 42]). Several other methods such as pyrolysis–gas chromatography combined with mass spectrometry (Pyr-GC/MS), high temperature gel-permeation chromatography (HT-GPC) with IR detection, SEM–EDS and thermoextraction and -desorption coupled with GC/MS [24, 41, 43–45] have been developed. With Pyr-GC/MS, both the polymer composition of microplastics and organic additives (e.g. antioxidants and plasticisers) can be analysed simultaneously [24, 43].

The abundance of microplastics is commonly indicated as numerical or mass concentration: (1) for the sea surface layer as number or weight of items per area, (2) for the water column as number or weight of items per volume and (3) for sediments as number or weight of items per sediment area or sediment weight, referring to sediment wet (ww) or dry weight (dw) (see also Additional file 1: Tables S1, S2). Due to the non-standardised units of quantification, the comparison of different studies can be very difficult [11, 29, 46]. It has therefore been suggested that all studies should provide sufficient information to allow converting units, e.g. from items per area to items per volume [11]. Preferably, both numerical and mass concentration should be indicated [8].

### Lower size limit of the sampled microplastics

The lower size limit of microplastics sampled in the environment is obviously determined by the sampling and processing methods. Microplastics larger than 300 µm were sampled in most cases from seawater and microplastics larger than 500 µm from sediment [11]. When density separation with a subsequent filtration step is used, smaller particles can be retrieved from sediment. The smallest microplastics sampled from sediment had a diameter of 1 µm. With current state-of-the-art techniques it is most likely not possible to representatively sample and unequivocally identify microplastics with a size below 1–2 µm [11, 26].

### Quality control

Given that microplastics research is still in a relatively early stage, inter-laboratory comparisons of protocols for sampling, processing and analysis and certified reference materials are lacking [6, 8, 29, 37]. There is little information on the recovery rates of different sampling and processing methods [26]. Especially small microplastics have a strong tendency to adsorb to surfaces. Therefore, care has to be taken to avoid overlooking particles that adhere to the devices used to collect and process samples [11, 26].

A further important issue is potential contamination of samples by particles originating e.g. from the clothing of workers, the used equipment and the ambient air. As far as possible, such contamination has to be reduced [6, 11, 26, 34, 47]. It has also been suggested that samples should be processed in cleanrooms or cleanroom cabinets and that procedural controls should be included to verify contamination during sample processing [6, 11, 28, 47–49].

### Summary: methods to sample, process and analyse microplastics in the environment

Appropriate sampling, extraction and identification methods are required to representatively sample and unequivocally identify microplastics in the environment. Visual sampling and sorting of small particles (<0.5–1.0 mm) and visual identification of microplastics are not considered reliable, since particles may be overlooked or wrongly classified as microplastics. Quality controls have to be included to verify the efficiency of the used methods and the absence of background contamination.

## Sources of microplastics and routes of entry into the environment

Microplastics found in the environment are a very heterogeneous group of particles differing in size, shape, chemical composition and specific density that originate from a variety of different sources. The following two sections provide an overview of sources of primary and secondary microplastics in the environment (see also Table 2). Primary microplastics are commonly defined as microplastics produced (and released to the environment) in a micro-size range; secondary microplastics result from the fragmentation of larger plastic materials [8, 50].

**Table 2:** Overview of sources for primary and secondary microplastics in the environment

Primary microplastics
Specific personal care products containing microplastics as exfoliants/abrasives
Specific medical applications (e.g. dentist tooth polish)
Drilling fluids for oil and gas exploration
Industrial abrasives
Pre-production plastics, production scrap, plastic regranulate: accidental losses, run-off from processing facilities
Secondary microplastics
General littering, dumping of plastic waste
Losses of waste during waste collection, from landfill sites and recycling facilities
Losses of plastic materials during natural disasters
Plastic mulching
Synthetic polymer particles used to improve soil quality and as composting additive
Abrasion/release of fibres from synthetic textiles
Release of fibres from hygiene products
Abrasion from car tyres
Paints based on synthetic polymers (ship paints, other protective paints, house paint, road paint): abrasion during use and paint removal, spills, illegal dumping
Abrasion from other plastic materials (e.g. household plastics)
Plastic items in organic waste
Plastic coated or laminated paper: losses in paper recycling facilities
Material lost or discarded from fishing vessels and aquaculture facilities
Material lost or discarded from merchant ships (including lost cargo), recreational boats, oil and gas platforms

Based on [9, 21, 25, 31, 53, 55, 57, 58, 60, 61, 70, 72, 75, 77–79, 81]

### Primary microplastics

Microplastic particles are used as exfoliants in certain product segments of specific personal care products, such as hand cleaners, facial cleaners and toothpaste [51]. In the US patent for skin cleaners containing plastic microparticles, polyolefin particles with a size of 74–420 µm and an amorphous shape without sharp edges were described as appropriate for use as exfoliants [52]. The used polyolefins include polyethylene (PE), polypropylene (PP) and polystyrene (PS; for abbreviations for the plastic resins see also Table 1). Gouin et al. [53] estimated that in 2012, approx. 6 % of the liquid skin cleaning products marketed in the European Union, Norway and Switzerland contained microplastics. Based on a survey conducted by Cosmetics Europe, PE accounted for 93 % of the microplastics used in skin cleaning products in these countries in 2012. The products typically contained between 0.05 and 12 % of microplastic particles, with the size of most particles ranging from 450 to 800 µm [53]. When analysing skin cleaners, spherical particles (mostly with a rough surface), threads and irregularly shaped particles consisting of PE and PS, and mainly having a blue or white colour were identified [45, 51, 54–56]. Microplastics are also used in medical applications, e.g. in dentist tooth polish, and as carriers to deliver active pharmaceutical agents [51, 57]. After use, microplastics from personal care products and such medical products can reach the environment via wastewater.

Microplastics are also used in drilling fluids for oil and gas exploration and in industrial abrasives, i.e. for air-blasting to remove paint from metal surfaces and for cleaning different types of engines [55, 57–59]. Industrial abrasives contain e.g. acrylic, PS, melamine, polyester (PES) and poly allyl diglycol carbonate microplastics [41]. If not used in closed systems and disposed properly, they can end up in the environment [57].

Raw materials used for the fabrication of plastic products (pre-production plastics), namely plastic resin pellets or flakes and plastic powder or fluff, are another important source of primary microplastics. They can reach the environment after accidental loss during transport or with run-off from processing facilities, i.e. often as a result of improper handling. Similarly, residues from plastic processing factories and regranulate produced during plastic recycling can end up in the environment [9, 25, 58–62]. Concentrations of plastic resin pellets in the environment were high from the 1970s to the 1990s [63, 64]. Highest concentrations of pre-production pellets (up to 100,000 pellets/m of beach) were often found on beaches close to plastic producing or processing sites [29, 60, 61]. In subsequent years, concentrations of pre-production plastics in the environment generally declined, probably due to improved practice during handling [63, 65, 66]. Still, high concentrations have been found in some studies close to production facilities ([67–69]; see also section “Occurrence of microplastics in the aquatic and terrestrial environment”).

### Macroplastics as sources of secondary microplastics

Since secondary microplastics are generated when larger plastic materials fragment, sources of macroplastics and their routes of entry into the environment are considered in this section. It has been estimated that about 75–90 % of the plastic debris in the marine environment originates from land-based and about 10–25 % from ocean-based sources [25, 70]. General littering, dumping of plastic waste and loss from inappropriately managed landfill sites and during waste collection are assumed to be the most important routes of entry of plastic materials into the environment. Windblown litter is also lost from recycling facilities [60, 70–72]. In this context, it should be noted that a large percentage of the produced plastics is used for packaging, i.e. for products with a short service life [1]. In industrialised countries, waste that is deposited in landfills is usually covered regularly with soil or a synthetic material, and the landfill is surrounded by a fence to prevent that debris is blown away. However, in developing regions this is often not the case [71–73]. In addition, large amounts of plastic debris can enter the marine environment during natural disasters such as hurricanes, tsunamis and strong sea [18, 74, 75].

Low-density polyethylene (LDPE) films, which are used in large volumes to protect agricultural crops, suppress weeds, increase temperature and retain irrigation water in the soil (‘plastic mulching’), are a further relevant source of microplastics in the environment. If these thin plastic foils embrittle, the fragments can end up in the soil [72, 76, 77]. Synthetic polymer particles, such as expanded PS flakes with a size of approx. 5–15 mm (Styromull^®^) and polyurethane (PU) foam, are also used in horticulture to improve soil quality and as composting additive [78, 79].

Moreover, synthetic textiles are an important source of microplastics. In 2013, 54.4 million t of synthetic fibres were produced worldwide with PES (44.6 million t) being the dominant fibre type [80]. Browne et al. [81] quantified the number of fibres released when washing PES blankets, fleeces and shirts in domestic washing machines. The washing machine effluent contained approx. 120 (blanket) to 300 fibres (fleece) per L. Overall, >1900 fibres were given off from the evaluated PES fleece during a single wash. Synthetic textile fibres are also released to air and dust, either during normal use [57] or during tumble drying [77]. In addition, synthetic fibres are released from hygiene products, e.g. if improperly disposed into wastewater [31].

A number of other sources of microplastics in the environment have been identified. Abrasion from car tyres has been considered as very relevant [46, 57]. In addition, many ship paints and other protective paints contain synthetic polymers, e.g. alkyds, poly(acrylate/styrene), PU and epoxy resins. Microplastics may be released by spills during application of the paint, by abrasion during use of the painted product and during paint removal [21, 57]. Microplastics are also released as a consequence of abrasion from other plastic materials such as household plastics [31, 57].

Ocean-based sources of marine litter include material lost or discarded from fishing vessels, aquaculture facilities, merchant ships, recreational boats, offshore oil or gas platforms and during military activities. Cargo lost from merchant ships may lead to a significant input of plastics into the marine environment [25, 58, 60, 72, 82]. Although dumping of plastic wastes at sea is prohibited since 1988, there are indications that plastic waste from a considerable number of vessels has still been dumped at sea—mainly due to economic reasons [46, 58, 83].

## Fate of microplastics in wastewater treatment plants

Primary and secondary microplastics as well as macroplastics may enter the environment through wastewater. During the primary (mechanical) treatment step in wastewater treatment plants (WWTPs), coarse suspended or floating solids are removed from the wastewater by screens or sieves. Sand and other heavy particles are retained in sand traps; floating material is removed in grease separators [84]. Coarse screens have openings of approx. 20–50 mm, intermediate screens of approx. 10–20 mm and fine screens of approx. 2–10 mm [84, 85]. Such screens are suitable for removing macroplastics from wastewater [72, 86, 87], while they will—based on the opening sizes mentioned above—not be able to capture smaller microplastics. Still, microplastics may be captured, if other materials are clogging the screens. Mintenig et al. [31] suggested that buoyant microplastics may be removed in the grease separating step. Microplastics with a high density such as PU can be expected to sediment and, thus, to be captured in the sand trap or with the sludge.

To date, only relatively few (often preliminary) studies are available on the effectivity of WWTPs to remove microplastics from wastewater, and on microplastic levels in WWTP effluents and sludge. In none of these studies, personal care products were unambiguously identified as source of the detected microplastics. This is due to the facts that (a) microplastics used in personal care products mostly consist of PE [53], the most widely used plastic resin [2], and (b) the often amorphous, irregular form of microplastics originating from such products is not typical enough to allow an identification of their source.

Effluents of two Australian WWTPs with tertiary treatment contained on average 1 microplastic item/L as identified by FT-IR. Polyester, acrylic and polyamide (PA) fibres were most frequently found [81]. Higher numbers were found in effluents from a German municipal WWTP (on average 33 granules/L, 24 fragments/L and 24 fibres/L [88]) and from three Dutch WWTPs (on average 55 microplastics/L [89]). However, in these two studies microplastics were only identified by visual analysis. In a Dutch pilot study, Leslie et al. [90] investigated microplastic concentrations in influent and effluent of a WWTP. With approx. 200 items/L in the WWTP influent and 20 items/L in the effluent of the activated sludge treatment, removal efficiency of the WWTP was approx. 90 %. A preliminary study was also performed in the central WWTP of St. Petersburg (Russia). Presumptive microplastic particles and fibres were identified by light microscopy. Approximately 95 % of fibres and particles present in WWTP influent were removed during wastewater treatment. The WWPT effluent contained on average 16 textile fibres, 7 coloured particles and 125 black particles/L [91].

A more comprehensive study was performed by Mintenig et al. [31] for 12 German WWTPs. Microplastics (0.02–5 mm) were analysed in WWTP effluents (sampled before final filtration, where present) and sewage sludge. Microplastics were identified by FT-IR or micro-FT-IR. In the WWTP effluents, 0.08–8.9 microplastic particles with a size <500 µm were detected per L. Particles with a whitish/transparent colour and an irregular and, partly, foil-like form were most common. The most frequently detected polymers were PE, polyvinyl alcohol, PES, PS and PA. The number of microplastic particles >500 µm ranged from 0 to 0.05 per L of effluent. Again, most particles had an irregular, partly foil-like shape. PE and PP were the most frequently found polymers. Plastic fibre content of the effluents ranged from 0.1 to 4.8 fibres/L. Effluent from one WWTP equipped with a final filtration step was also sampled after filtration. Filtration removed all microplastic particles >500 µm, 93 % of the microplastic particles <500 µm and 98 % of the microplastic fibres. Sewage sludge contained 1041–24,129 microplastic particles/kg dw (fibre content was not analysed). Since single samples were investigated for each WWTP and matrix and small subsamples were evaluated for sludge, Mintenig et al. [31] point out that their results should be considered as indicative values.

Size and form of microplastic fibres in sewage sludge can be affected during sludge stabilisation, e.g. as a consequence of mechanical mixing, increased temperature and increased pH [92, 93]. Sewage sludge is incinerated, disposed of in landfills or used to fertilise agricultural land [93], i.e. can represent a source of microplastics for the terrestrial environment. Microplastics may remain in the soil, be mobilised and distributed by wind, or be transported with surface run-off to the aquatic environment [31, 77, 94, 95]. When sewage sludge is disposed into oceans, microplastics directly reach the aquatic environment. In most industrialised countries, ocean disposal of sewage sludge is prohibited. However, in some countries sewage sludge is still disposed at sea [96].

During heavy rainfall events, sewer overflow may occur, i.e. untreated wastewater may reach the environment. Sewer overflow events have been assumed to be relevant with regard to the entry of microplastics into the environment [46, 72]. Furthermore, untreated sewage is in many regions of the world directly discharged into the receiving waters [82]. In the OECD countries, wastewater of approx. 80 % of the population is discharged to WWTPs [97]. However, worldwide only about 15–20 % of wastewater is treated [98].

## Relevance of different sources of microplastics in the environment

In most cases, it is not possible to derive conclusions on the origin of microplastics when evaluating and characterising their occurrence in an environmental compartment. So far, the contribution of specific sources has only been identified for microplastics with a typical and distinct size and shape. Examples include pre-production plastic resin pellets, especially if released by localised spills [60, 63, 99, 100], and styrofoam particles found close to intensive aquaculture facilities, where styrofoam buoys and floats are used [8, 101, 102]. By contrast, microplastics from personal care products lack such distinct characteristics that would allow an unequivocal identification of their source as has been discussed in the previous section.

Based on produced or consumed amounts, estimates have been derived on the contribution of various sources to the overall amount of microplastic debris in the environment. However, such estimates are hampered by the complexity of the sources of micro- and macroplastics, the lack of quantitative data on transport and fate in the environment (see next section), and the high geographic variability of the relevance of different sources and introduction routes, which is caused by differences in the infrastructure, especially with regard to waste management [70, 72, 73, 99, 103].

Consequently, quantitative information on the contribution of different sources to the overall amount of macro- and, especially microplastics in the environment is generally lacking [8, 104, 105]. There are, e.g. no reliable estimates of the percentage of plastic packaging material reaching the marine environment [25]. Given the large amount of macroplastics entering the environment, it is generally assumed that most microplastics in the environment are secondary microplastics, i.e. a result of weathering of larger plastic debris [11, 25]. However, fragmentation rates of macroplastics are largely unknown [57, 59, 99]. As a result, no quantitative information is available on the relative contribution of primary and secondary microplastics to the overall amount of microplastics in the environment [50].

A first estimate of the relative contribution of microplastics from personal care products to the plastic debris entering the North Sea has been provided by Gouin et al. [53]. Based on sales data for liquid skin cleaning products and the estimates that (a) 6 % of liquid skin cleaners contains microplastic particles and (b) these products contain 10 % of microplastics, a mean annual amount of 4130 t of microplastic particles was derived for the European Union, Norway and Switzerland for 2012. This value is consistent with the result of the previously mentioned survey of Cosmetics Europe (4360 t for the same region and year). For the countries in the watershed of the North Sea (Norway, Denmark, Germany, Belgium, The Netherlands, France, Switzerland, Czech Republic and the UK), annual use of microplastics in personal care products was estimated to be 2,300 t. Assuming removal of 90 % of the microplastics in WWTPs [90] and discharge of all water from these countries to the North Sea, microplastics from personal care products would constitute approx. 1 % of the overall amount of marine debris that has been estimated to enter the North Sea each year (20,000 t [106]). Since it is not specified how the amount of 20,000 t was estimated (see also [107]), the abovementioned information on the relative contribution of microplastics to the overall amount of plastic debris in the North Sea should be considered as a very rough estimate.

Sundt et al. [57] evaluated the most relevant sources for direct release of microplastics to the Norwegian environment. Such ‘primary sources’ of microplastics exclude macroplastic litter, but include abrasion of microplastics (e.g. from paints and tyres), i.e. are not confined to primary microplastics as defined in the present review. As far as possible, first estimates were provided for annually released amounts. These amounts are upstream or ‘start of the pipe’ amounts; transport processes are not considered. The estimated annually used amount of microplastics in personal care products (40 t) is in good agreement with the estimate of Gouin et al. [53] for Norway (43 t). According to Sundt et al. [57] microplastics from personal care products account for approx. 0.5 % of all direct emissions of microplastics in Norway. Other sources such as losses of pre-production plastics during transport and spills (approx. 5 %), abrasion from ship paints, other protective paints, house and road paints (approx. 17 %), release of textile fibres during household and commercial laundry (approx. 8 %) and, especially, abrasion from tyres (approx. 54 %) were considered more relevant. Sundt et al. [57] assumed that macroplastic litter substantially contributes to the overall release of microplastics to the Norwegian environment. However, the available data were not considered sufficient for deriving estimates of this contribution. First estimates were only provided for annual amounts of three types of macroplastic litter in Norway: plastic waste from fisheries and aquaculture (>1000 t), littered plastic bags (60 t) and macroplastics released during sewer overflow events (460 t). Estimates derived for Germany and Denmark also indicate that personal care products are a minor source of microplastics in the environment and that other sources such as the fragmentation of plastic debris and abrasion from tyres are more relevant [51, 59]. For Denmark, emissions of microplastics from personal care products to the aquatic environment were estimated to account for 0.1 % of the overall emissions to the aquatic environment [51].

Recently, a number of companies producing personal care products have announced a phase-out of microplastic particles in their products. In addition, several US states have banned the manufacture and sale of personal care products containing microplastic particles [53, 57, 59, 108, 109]. It can thus be assumed that at least for Europe and the USA emissions from personal care products will decrease in the near future. However, the issue of microplastics in the environment will certainly not be solved by these actions, since primary microplastics from this source only contribute a small percentage based the rough calculations mentioned above.

## Fate of macro- and microplastics in the environment

Once released into the environment, plastic material can be transported by wind, washed from land to surface waters during rainfall, especially with stormwater run-off, and be transported in freshwater and seawater [61, 63, 72]. It is assumed that large rivers transport considerable amounts of macro- and microplastics to the oceans, but few quantitative data are available [96, 110–112]. For macroplastics, transport by wind to the oceans may be significant, especially since approx. 50 % of the human population live within 80 km distance from the sea [9]. Airborne transport might also be relevant for very small microplastics, which could e.g. be mobilised from uncovered landfills [77].

Fate in the aquatic environment depends on the density: plastics with a lower density than freshwater (approx. 1.0 g/cm^3^) or seawater (approx. 1.03 g/cm^3^) are buoyant, those with a higher density are submerged [25]. Most consumer plastic materials (including PE) are buoyant in seawater (Table 1). In aquatic environments, plastics are often colonised by a variety of organisms. This fouling can increase the density so that formerly buoyant items sink below the water surface. If other organisms graze on the foulants, density of a plastic item can decrease again so that it returns to the water surface [25, 71]. Erosion of plastics may also change their specific density [66], and mixing of the upper water layer by wind may lead to submersion of previously buoyant microplastics [19].

Given that plastic is persistent (see below) it can be transported over long distances, depending on local winds, ocean currents and geography of the coastline [11, 71]. Floating plastic debris accumulates on beaches and in oceanic gyres, benthic debris on the sea bed in areas with low circulation [9, 11, 71]. The transport of microplastics in surface waters may differ from that of macroplastics, because smaller particles at the water surface are less exposed to wind. Distribution of microplastics may also be affected by particle aggregation and activity of animals [113, 114]. On beaches and in subtidal sediments, microplastics can be covered by sediment [11] and, hence be found at considerable depth [115].

So far, little is known on the transport of microplastics within the terrestrial environment. Rillig [77] assumed that terrestrial organisms such as earthworms and moles contribute to the incorporation of micro- and macroplastic material into the soil.

Disintegration of common polymers such as LDPE, high-density polyethylene (HDPE) and PP in the environment is mainly initiated by UV radiation. As a result of this photo-oxidative degradation, the plastic becomes brittle and fragments. Disintegration is facilitated by increased temperatures, but reduced by stabilisers, low temperature, low oxygen levels and by fouling or coverage with water or sediment reducing exposure to UV radiation. Hence, photo-oxidative disintegration of plastics is relatively effective on a beach surface, but extremely slow in the deep ocean [25, 71, 116] and for plastics buried in beach sediment or soil [72, 82]. Water turbulences, wave action, physical abrasion and freeze–thaw cycles add to the disintegration of the plastics [116]. In addition, the boring activity of isopods substantially contributed to the fragmentation of expanded polystyrene (EPS) floats used in aquaculture facilities and docks [117]. Similarly, soil organisms that ingest plastic debris together with earth could contribute to the fragmentation of the plastics [77].

During disintegration of the polymer matrix, particles of different sizes and shapes are formed [11, 71, 72]. Note that strongly weathered, brittle plastic material still consists of polymers with a mean molecular weight of tens of thousands g/mol, which are not biodegraded to considerable extent under relevant environmental (especially marine) conditions [25]. Based on the data available so far, mineralisation of plastics appears to be an extremely slow process [25, 116]. For example, sheets of LDPE, HDPE and PP that had been immersed for 6 months in sea water only lost 1.5–2.5 % (LDPE), 0.5–0.8 % (HDPE) and 0.5–0.6 % (PP) of their initial weight [118]. Consequently, estimates of the lifetime of plastics are in the range of hundreds of years [9, 71]. It has therefore been assumed that all conventional plastic, which has entered the environment, is still present in the environment, either in an unfragmented or in a fragmented form [74].

## Occurrence of microplastics in the aquatic and terrestrial environment

### Marine environment

Triggered by the detection of unexpectedly high levels of microplastics in oceanic convergence zones (e.g. [3, 25, 119]), the occurrence of microplastics has mainly been investigated in the marine environment (including shorelines). Microplastics have been found in the oceans worldwide [71, 120], including remote regions such as Antarctica and the deep sea [58, 121–124]. The distribution of microplastics in the oceans is very heterogeneous. High concentrations have been found close to industrial centres and metropolitan areas [22, 58, 110, 125], in enclosed or semi-enclosed seas such as the Caribbean and the Mediterranean Sea and in gyres [8, 64, 71, 126].

Clear increases in the abundance of microplastics in seawater have been found over larger temporal scales. Thompson et al. [127] evaluated archived plankton samples collected with continuous plankton recorders in the northeast Atlantic at 10 m depth. Samples were examined microscopically and unusual fragments were identified by FT-IR. The concentration of microplastics significantly increased from the 1960s and 1970s (approx. 0.01 items/m^3^) to the 1980s and 1990s (approx. 0.04–0.05 items/m^3^). Based on published data and own investigations using a manta trawl and visual identification of microplastics Goldstein et al. [128] evaluated temporal changes in microplastic levels in the surface layer of the North Pacific gyre. They found that between 1972–1987 and 1999–2010 the median numerical concentration of microplastics had increased by a factor of approx. 140 (from 0.003 to 0.425 items/m^3^) and the median mass concentration by a factor of 1000 (from 0.003 to 3 mg/m^3^). Law et al. [20] re-analysed the temporal trend using a combined dataset including the data of Goldstein et al. [128] and their own data obtained by sampling using neuston nets and visual identification of small plastic items (typically mm-sized; see Additional file 1: Table S1). To reduce the bias, which is caused by an increased sampling frequency in areas with high plastic levels in recent years, they calculated average concentrations of small plastic items for each 1° × 1° (latitude × longitude) area. On the basis of these data, Law et al. [20] concluded that both mean and median numbers of small plastic items in the surface layer of the North Pacific gyre increased by a factor of approx. 10 between 1972–1985 and 2002–2012. It is controversially discussed if the increasing trend has continued since the 1990s [20, 65, 71, 128, 129]. Due to large spatial and temporal variability in the concentrations of microplastics, it is difficult to detect smaller increases [19]. High variability of the levels of small plastic items at a smaller scale (i.e. within tens of km) is caused by local wind-driven turbulences and local circulation patterns [20]. Higher concentrations in the sea surface layer are e.g. measured during low-wind conditions [19, 126]. Temporal variability can be high, e.g. due to variations in oceanic circulations related to El Niño events [71, 99]. Due to the embrittlement and fragmentation of larger plastic items present in the oceans, it has been predicted that the overall abundance of microplastics will increase in the future [25, 71, 99, 120].

In the following, an overview is provided of the concentration ranges of microplastics in the marine environment (including estuaries) that is based on the review of Hidalgo-Ruz et al. [11] and selected recent publications. The compiled data shall primarily serve as background information when evaluating the environmental relevance of test concentrations in studies on uptake and effects of microplastics. For this reason, main focus is placed on studies reporting numerical concentrations per water volume, sediment weight or sediment volume. As outlined above, most data on the occurrence of microplastics in the marine environment have been obtained using visual selection and often also visual identification of microplastics [11]; the results of quality controls are only reported in some cases (e.g. [49, 110, 123]). Although data obtained by visually selecting and/or identifying small microplastics should be considered with care, they provide a first impression on the abundance of microplastics in marine systems and were, thus, not excluded from the present review. However, an attempt was made to especially include recent publications using state-of-the-art sampling, extraction and identification techniques (see Additional file 1: Table S1 for information on the used methods).

As most consumer plastics are at least initially buoyant in seawater, the abundance of microplastics in the sea surface layer, the upper approx. 20 cm of the water column, was addressed in many studies. Buoyant micro- and macroplastics have been found to accumulate in the North Atlantic, South Atlantic, North Pacific, South Pacific and Indian Ocean gyre [65, 119, 128–130]. In the North Pacific gyre, mean abundance of visually identified plastic items (0.33 items/m^2^, mostly fragments, plastic films and fibres with a size <5 mm) in 3 out of 11 samples was found to be higher than plankton abundance [119]. Based on the review of Hidalgo-Ruz et al. [11] and the evaluated recently published studies (Additional file 1: Table S1), concentrations of microplastics in the sea surface layer range from 0 to 12.3 items/m^2^ [20] and from 0 to approx. 8700 items/m^3^ (i.e. 8.7 items/L [11]). A much higher abundance of synthetic polymer particles was found in the sea surface microlayer, i.e. the upper 1 mm of the water column (Additional file 1: Table S1). Mean abundance was 195 items/L for paint particles, mainly alkyd and poly(acrylate/styrene) polymers and 16 items/L for non-paint microplastics [21].

In the water column below the sea surface layer, concentrations of microplastics can be expected to be lower than at the water surface. According to the evaluation of Hidalgo-Ruz et al. [11], concentrations of microplastics in the water column ranged from 0.014 to 12.5 items/m^3^. Considerably higher concentrations were detected in a recent study [18] in the north-eastern Pacific and close to the coast of British Columbia (Canada). Concentrations of microplastics in seawater sampled from a depth of 4.5 m ranged from 8 to 9180 items/m^3^. They were lowest at offshore sites in the north-eastern Pacific (mean: 279 items/m^3^) and higher at nearshore sites (1710–7630 items/m^3^). Approx. 75 % of the microplastics were fibres; the percentage of fibres increased near the shores. As noted by the authors, fibre content was underestimated: brightly coloured brittle fibres were observed in most samples, but could not be quantified since they were eliminated during acid digestion. High concentrations of microplastics (0.5 mm to approx. 5 mm) were also found in the Yangtze estuary in China [131]. In water samples collected at 1 m depth, the average concentration of microplastics was 4137 items/m^3^.

Coastal and, especially, subtidal sediments appear to be sinks for microplastics [89, 115, 123]. As pointed out by Hidalgo-Ruz et al. [11] microplastic concentrations in sediments tend to be much higher than those in the sea surface layer and in the water column. Microplastics have been detected in coastal sediments (in most cases beaches) around the world [81, 99]. At some sites, they accounted for 80 % of the intertidal plastic debris [132].

Microplastic levels vary greatly between beaches. Claessens et al. [110] and Van Cauwenberghe et al. [105] evaluated microplastics (≤1 mm) on Belgian beaches. Claessens et al. [110] recorded an average concentration of 93 items/kg sediment dw with plastic fibres accounting for 88 % of the microplastics, granules for 7 % and plastic films for 5 %. Van Cauwenberghe et al. [105] found a mean concentration of 13 items/kg sediment dw. In both studies, highest microplastic levels were observed at the high tide line, where resuspension of particles during flooding occurs less frequently. In sediments from a beach on the East Frisian Island Norderney (Germany), levels of microplastic particles (<1 mm) were low (mean values: 1.7, 1.3 and 2.3 particles/kg sediment dw). Due to background contamination with fibres, fibre content of the sediment was not evaluated [49].

Much higher concentrations of microplastics (at least 2 dimensions <5 mm) were found on beaches of three Canary Islands [133]. They ranged from 1 to 30 (Fuerteventura), <1–109 (Lanzarote) and <1–90 g/L sediment (La Graciosa). Low microplastic levels were found on rocky shores and on beaches being completely submerged at high tide (i.e. offering no space for deposition of plastics). Highest levels were recorded on the northern coasts, which are reached by currents likely to transport plastic debris. Carson et al. [134] detected partly extremely high levels of small plastic particles (<10 mm) on southern Hawaii, on two beaches located close to the North Pacific subtropical convergent zone. Kamilo beach sediment contained on average 1.3 % (w/w) of small plastic particles. More than 50 % of the particles were found in the upper 5 cm of the sediment, which contained on average 3.3 % (w/w) of small plastics (the maximum value was 30 %). At Waikapuna beach, mean plastic content was 0.03 %. Again, most plastic occurred in the top 5 cm of the beach, where plastic content was 0.1 %. The plastic particles analysed by FT-IR mainly consisted of PE (85 %) and PP (14 %). Elevated concentrations of small plastic particles (mean: 805 items/m^2^) were also found on beaches on the Easter Islands [101]. Lee et al. [102] recorded high microplastic levels (mean values: 8205 items/m^2^ before and 27,606 items/m^2^ after the rainy season) on South Korean beaches close to the estuary of the Nakdong River, which flows through a densely populated metropolitan area.

Microplastic levels in subtidal sediments from harbours, coastal and offshore areas of the Belgian continental shelf were investigated by Claessens et al. [110]. Harbour sediments contained significantly more microplastics (mean: 167 items/kg sediment dw) than coastal (92 items/kg sediment dw) and offshore sediments (105 items/kg sediment dw). In harbour sediments, fibres accounted for 40 % of the microplastics, granules for 34 %, plastic films for 4 % and PS spheres for 22 %. Microplastics in coastal and offshore sediments consisted of 68 % fibres, 30 % granules and 2 % plastic films. High concentrations in harbour sediments were thought to be related to local input and to the fact that the studied harbour areas were partly enclosed. The analysed fibres consisted of PA, polyvinyl alcohol and PP, the granules of PS, PE and PP. Concentrations of microplastics (<1 mm) in sediments sampled at approx. 1 m depth in the Lagoon of Venice (Italy) were studied by Vianello et al. [22]. Levels of microplastics ranged from 672 to 2175 items/kg sediment dw. They were higher in the inner area of the lagoon, where water currents were low (i.e. more sedimentation occurred). Most microplastics consisted of PE (48 %) or PP (34 %). Irregularly shaped fragments accounted for 86 % of all microplastics, fibres for 11 %, films for 2 % and pellets/granules for 1 %.

In an early study using polarised light microscopy and semi-quantitative evaluation, Habib et al. [92] found that abundance and size of textile fibres in subtidal sediments decreased with increasing distance from a WWTP. Browne et al. [81] sampled subtidal sediments from two sites, where sewage sludge had been disposed, and two reference sites (Additional file 1: Table S1). Although disposal of sewage sludge had been stopped more than 10 years before, the former disposal sites contained 4 times (North Sea) and 2.5 times (English Channel) more microplastics than the reference site.

Microplastics were also detected in deep sea sediments, e.g. in the northeast North Atlantic and southwest Indian Ocean and in the Mediterranean Sea at water depths between 300 and 3500 m [123]. The sediments contained 28–800 microplastics/L. Notably, exclusively fibres were found. Likewise, fibres were the dominant type of microplastics found in sediments from the Kuril-Kamchatka Trench (northwest Pacific) at water depths of 4869–5766 m. In addition, paint chips and fragments were recorded. The microplastics did not only occur in the upper 2 cm of the sediment, but also in deeper sediment layers [124].

### Freshwater environment

So far, there are only relatively few studies on microplastics in the freshwater environment. These studies have focused on larger rivers and lakes, while there are no data on the occurrence of microplastics in smaller streams or lakes [72, 95, 111].

During a 2-year survey, Lechner et al. [68] evaluated the abundance of small plastic items (0.5–20 mm) in the surface layer of River Danube between Vienna (Austria) and Bratislava (Czech Republic). In 2010, mean concentration of small plastic items was 0.938 items/m^3^; 86 % of these items were pre-production plastics. In 2012, plastic abundance was much lower (0.055 items/m^3^); pre-production plastics accounted for 31 % of these items. The high levels of pre-production plastics in 2010 were apparently caused by leakages in the pipe system of a plastic producer and by a strong rain event, during which pellets were washed into the Danube [135–137]. Recently, a similar mean microplastic concentration (0.29 items/m^3^) was derived for the surface layer of the River Rhône [138]. The microplastics mainly consisted of fragments (40 %), foams (37 %) and fibres (14 %). A clear effect of a municipal WWTP on microplastic levels was demonstrated in North Shore Channel (Chicago, USA). Mean microplastic concentration in the surface layer increased from 1.94 items/m^3^ upstream of the WWTP to 17.93 items/m^3^ downstream of the WWTP [139]. In sediments from St. Lawrence River (Canada), plastic granules with diameters of 0.4–2.2 mm and mainly grey or black colour were detected. Based on their melting point, it was assumed that the granules consist of PE. Personal care products were mentioned as possible source. However, highest concentrations (136,926 items/m^2^) were found in the effluent canal of a nuclear power plant, while concentrations at the other nine sampling sites ranged from 0 to 243 items/m^2^ [140]. This suggests that the granules may originate from a source other than personal care products that remains to be identified.

In a preliminary study of microplastic (0.3–5 mm) levels in the surface layer of Lake Geneva (Switzerland), a microplastic concentration of 0.048 items/m^2^ was derived [141]. A similar mean microplastic level (0.091 items/m^2^) was determined in six lakes located (or partly located) in Switzerland [138]. Fragments, foams, films and fibres were dominant. Microplastics (0.355 to approx. 5 mm) were also analysed in three of the Great Lakes: Lake Superior, Lake Huron and Lake Erie [41]. The mean concentration in the surface layer of these lakes was 0.043 items/m^2^; 81 % of all microplastics had a size ≤1 mm. The highest microplastics levels (0.463 items/m^2^) were detected in Lake Erie, downstream of the cities of Detroit, Cleveland and Erie. Fragments, pellets and foams were found most frequently. The particles also included green, blue and purple spheres, which had a similar size, shape, colour and elemental composition as microbeads from facial cleansers analysed by Eriksen et al. [41]. However, more specific information on the abundance of the coloured spheres in the surface layer from the Great Lakes is not provided. Only slightly lower levels of microplastics (0.355 mm to approx. 5 mm) were recorded in the surface layer of Lake Hovsgol, a large mountain lake in northern Mongolia [103]. Fragments, films and lines/fibres were the dominant items. The average microplastic concentration was 0.0203 items/m^2^. Around the lake, there is no industry, and population density is low. Free et al. [103] assumed that the complete lack of a waste management system (waste is burned, buried or dumped) and of wastewater treatment are the causes for the relatively high microplastic levels. Microplastic concentrations were highest in the most populated and most touristic part of the lake. A parallel survey of macroplastic showed that household plastics (plastic bottles and bags) and fishing gear were the dominant macroplastic items at the lake shores.

In sediments sampled at the shores of the rivers Rhine and Main in Germany, microplastic concentrations of 228–3763 items/kg dw (River Rhine) and 786–1368 items/kg dw (River Main) were recorded with PE, PP and PS being the dominant polymers [112].

Imhof et al. [142] evaluated levels of microplastics (<5 mm) in sediment samples from two beaches of Lake Garda (Italy). Microplastic concentration was much higher at the northern (1108 items/m^2^) than at the southern lake shore (108 items/m^2^). This difference was attributed to the prevailing wind direction and the resulting water circulation. PS, PE and PP particles were most frequently found. With regard to the smallest microplastics (<500 µm), PA and polyvinyl chloride (PVC) were also relevant. Most particles were classified as fragments; signs of degradation/fragmentation were identified by SEM. At beaches of six lakes in Switzerland, microplastic levels ranged from 20 to 7200 items/m^2^. Foam, fragments and fibres were found most frequently [138].

### Terrestrial environment

Although microplastics obviously enter the terrestrial environment (e.g. due to littering and application of sewage sludge to land) and soils have been assumed to be a sink for microplastics, there are only extremely few data on microplastic concentrations in the terrestrial environment [71, 72, 77]. It was suggested to use synthetic fibres as indicators of previous sludge application to land [92, 93]. Using polarised light microscopy, Zubris and Richards [93] showed that up to 15 years after sludge application, levels of plastic fibres in soil of a long-term experimental field site clearly exceeded levels at control sites.

### Summary: occurrence of microplastics in the aquatic and terrestrial environment

As a consequence of their persistence and long-range transport, microplastics are ubiquitous in the marine environment. Notably, beaches (where extremely high levels have been recorded at some cases) and subtidal sediments appear to be sinks for microplastics. Based on first investigations, microplastic levels in large rivers and lakes are similar to those in the marine environment. The occurrence of microplastics in smaller streams and lakes and, especially, in the terrestrial environment remains to be investigated.

In aquatic environments, fibres, fragments, granules, films and styrofoam particles are the most commonly found particle types, while PE, PP and PS are the most frequently found polymers. Due to the lack of distinct characteristics, it is generally not feasible to unequivocally identify personal care products as source of microplastics detected in the environment.

## Uptake of microplastics by organisms in the environment and trophic transfer

Due to their low size, microplastics are ingested by a variety of species ranging from protozoans to marine mammals [7, 75, 143, 144]. Their uptake depends on properties such as size, shape, density and colour. For instance, low-density (i.e. buoyant) microplastics are ingested by pelagic filter feeders, high-density microplastics by benthic deposit feeders [7, 132, 145]. Many filter feeding and deposit feeding organisms are indiscriminate feeders: they capture food of a suitable size without further selection [9]. The following four sections provide an overview of uptake, translocation within the body, excretion and trophic transfer of microplastics as investigated in laboratory studies with aquatic organisms. Afterwards, field studies with aquatic organisms and studies with terrestrial organisms are discussed.

### Uptake by aquatic organisms

In laboratory experiments, ingestion of microplastics has been demonstrated for a number of marine invertebrates including ciliates, cnidarians, rotifers, annelids, copepods, cladocerans, amphipods, mysids, euphausiids, barnacles, mussels and tunicates [8, 127, 145–147] and fish [148]. For example, Lee et al. [149] investigated the uptake of nano- (50 nm) and microsized (0.5 and 6 µm) fluorescently labelled PS spheres by the copepod *Tigriopus japonicus*. Copepods were exposed for 24 h to concentrations of 9.1 × 10^14^ (50 nm), 9.1 × 10^11^ (0.5 µm) and 5.3 × 10^8^ items/L (6 µm). Spheres of all three sizes were detected in the gut of the copepods. In the marine amphipod *Allorchestes compressa*, which had been exposed for 72 h to a very high concentration (100 g/L) of PE microplastics (11–700 µm), on average 19 particles per amphipod were detected [150]. Ingestion of microplastics was also demonstrated in larvae of the sea urchin *Tripneustes gratilla* held for 5 d at concentrations of 10^3^–3 × 10^5^ items/L of fluorescent PE microspheres (10–45 µm). At the highest concentration up to 31 % of sea urchin larvae contained microplastics in their stomachs, at the lowest concentration up to 5 % (on average 1–2 microspheres per larva; [151]).

In addition to being ingested, small microplastics might also be taken up via the gills [8]. For mussels, it has been suggested that uptake via the gill surface (endocytosis) may be relevant for smaller microplastics, while larger particles are taken up via the digestive system ([152], see next section for further details on this study). In shore crabs (*Carcinus maenas*) exposed to PS microspheres (8–10 µm), exclusively via the ventilation route, microspheres were detected on the gill surface but not in the gill tissue [153].

Uptake of microplastics by freshwater organisms has so far only been addressed in relatively few studies. As to be expected, the available data show that microplastics are also ingested by freshwater organisms. Fluorescent PS microparticles (1 µm) were ingested by protozoans (*Paramecium* sp.) and *Daphnia* sp. [144]. Nano- (20 nm) and microsized (1 µm) fluorescent carboxylated PS spheres were taken up by neonates and adults of *Daphnia magna* [154]. Ground fluorescent polymethyl methacrylate particles (approx. 30 µm) were ingested by *D.* *magna*, the ostracod *Notodromas monacha*, the amphipod *Gammarus pulex*, the snail *Potamopyrgus antipodarum* and the oligochaete *Lumbriculus variegatus* [142].

### Transfer from the intestinal tract to the surrounding tissue or circulatory system

After ingestion, microplastics can remain in the digestive tract, be excreted or absorbed from the digestive tract into the body tissue [132, 145]. Lugworms (*Arenicola marina*) exposed to sediment containing pre-production PS particles (400–1300 µm, 7.4 % of sediment dw) ingested these microplastics. However, no translocation of the relatively large PS particles from the gut to the tissue was recorded [155]. Hämer et al. [156] fed marine isopods (*Idotea emarginata*) with food containing fluorescent PS microspheres (10 µm), PS fragments (1–100 µm) or acrylic fibres (0.02–2.5 mm). Microplastics were detected in stomach and intestine, but not in the midgut where nutrients are resorbed. Passage of the microplastics to the midgut was most likely impeded by filter structures in the isopods’ proventriculus. In *D.* *magna*, fluorescent-carboxylated PS nano- and microspheres (20 nm and 1 µm diameter) were mainly observed in the gastrointestinal tract, but also in structures assumed to be oil storage droplets. It was concluded that the PS spheres are able to cross the gut epithelium [154]. In shore crabs (*C.* *maenas*), which were fed with mussels (*Mytilus edulis*) pre-exposed to PS microspheres, translocation from the intestinal tract to haemolymph, hepatopancreas, ovary and gills was demonstrated for microspheres with 0.5 µm diameter [157]. By contrast, larger microspheres (8–10 µm diameter) were only detected in the intestine but not in the haemolymph of shore crabs [153].

Browne et al. [145] kept mussels (*M.* *edulis*) for 3 h in a suspension of fluorescent PS microspheres (3.0 and 9.6 µm; 4.3 × 10^4^ items/L). Microspheres were detected in the haemolymph and inside the haemocytes. The smaller microspheres occurred in significantly higher abundance in the haemolymph than the larger ones. Von Moos et al. [152] exposed mussels for 3–96 h to 2.5 g/L of HDPE fluff consisting of non-uniformly shaped particles with a size between 0 and 80 µm. The concentration of HDPE fluff corresponds to approx. 2.7 × 10^7^ to 3.6 × 10^7^ items/L (NR von Moos, personal communication). HDPE microparticles were detected on the gill surface and in blood lacunae of the gills, as well as in the intestine, digestive gland and connective tissue.

In a very recent study, mullets (*Mugil cephalus*) were held for 7 days in water containing 33.8 mg/L of PE or PS particles with a size of 0.1–1 mm (nearly 2500 particles/L [33]). Microplastics were not only found in the gastrointestinal tract (approx. 10 PE particles and 90 PS particles per fish), but also in the liver of the fish (approx. 1–2 particles per fish for both PE and PS).

Thus, based on the results of laboratory experiments, translocation from the intestinal tract to the circulatory system or surrounding tissue depends on the size of the microplastics with an upper size limit for translocation that appears to be specific for the species or taxonomic group.

### Excretion by aquatic organisms

In laboratory experiments, microplastics, which had only entered the organisms’ intestinal tract, were generally excreted within hours or few days. Rapid excretion of microplastics was e.g. reported for the copepod *Eurytemora affinis* [147]. After 3 h exposure to fluorescent PE spheres (10 µm; 2 × 10^6^ items/L), 67 % of the copepods had ingested microspheres. Following a 12 h post-exposure, only 4 % of the copepods still contained microspheres. In marine amphipods (*A.* *compressa*), most ingested PE microplastics were excreted within 2 d [150]. Sea urchin larvae (*T.* *gratilla*) egested PE microspheres (10–45 µm) within 7 h [151].

In cases, where microplastics had translocated to the circulatory system and/or the surrounding tissues, excretion was also demonstrated, but was slower. In shore crabs (*C.* *maenas*), concentrations of fluorescent PS microspheres (0.5 µm) in the haemolymph decreased from 24 h to 21 days post-exposure. Yet, on day 21 the haemolymph still contained a few microspheres [157]. In mussels (*M.* *edulis*), fluorescent PS microspheres (3.0 and 9.6 µm) were—despite excretion with the faeces—still detected in the haemolymph 48 d post-exposure [144].

### Trophic transfer

Several laboratory studies have demonstrated that microplastics are transferred in the food chain, i.e. from prey to predator. Trophic transfer of fluorescent PS microspheres (10 µm) from zooplankton to the mysid shrimp *Mysis relicta* was observed by Setälä et al. [147]. *M.* *relicta*, which had been kept together with zooplankton (copepods and polychaete larvae) pre-exposed for 12 h to microplastics, contained PS microspheres in their stomach. Trophic transfer of microplastics from mussels (*M.* *edulis*) to shore crabs (*C.* *maenas*) was shown for fluorescently labelled PS microspheres with 0.5 µm [157] and 8–10 µm diameter [153]. So far, it is not known if microplastics biomagnify, i.e. if concentrations in the organisms increase at higher trophic level [158].

### Field studies with aquatic organisms

The uptake of microplastics has also been investigated in a number of field studies, mainly with marine organisms. Note that in these studies no differentiation between direct ingestion of microplastics and uptake with the food (i.e. trophic transfer) is possible.

Desforges et al. [36] analysed microplastics in two zooplankton species, the copepod *Neocalanus cristatus* and the euphausiid *Euphausia pacifica*, sampled in the Northeast Pacific. On average, 0.03 microplastic items/individual were found in *N. cristatus*, 0.06 in *E.* *pacifica*. Goldstein and Goodwin [159] evaluated plastic ingestion by gooseneck barnacles (*Lepas anatifera*, *Lepas pacifica*, *Lepas* sp.), rafting organisms attached to floating substrates at the sea surface. Gooseneck barnacles were sampled from floating debris in the North Pacific gyre; microplastics in their stomachs and intestines were identified visually. Due to the possibility of airborne contamination, fine microfibers were not considered. A subset of microplastic particles was analysed by Raman spectrometry. Microplastics were found in 34 % of the barnacles, with most barnacles having ≤5 particles in their intestinal tract. Nearly all (99 %) particles were degraded fragments (median diameter: 1.4 mm). No blockage of the stomach or intestine was recorded. Analysis by Raman spectrometry revealed that 58 % of the particles consisted of PE, 5 % of PP and 1 % of PS (polymer type of the remaining particles could not be analysed).

Van Cauwenberghe and Janssen [38] investigated levels of microplastics in cultured mussels (*M.* *edulis*) reared in the North Sea and Pacific oyster (*Crassostrea gigas*) reared in the Atlantic. Mussels were either analysed directly for their microplastic content or kept for 3 days in filtered artificial seawater to allow clearing of the gut. Soft tissues were digested in concentrated nitric acid, boiled, diluted with deionised water and filtered (5 µm). Microplastics were identified microscopically, and a subset was analysed by micro-Raman-spectrometry. Samples were processed in a laminar flow cabinet; procedural controls were free of microplastics. In *M.* *edulis*, 0.36 microparticles per g soft tissue ww were detected without depuration. After the depuration period, 0.24 items/g soft tissue ww were found. In *C.* *gigas*, 0.47 items/g soft tissue ww were found before and 0.35 items/g tissue ww after depuration. As discussed by Van Cauwenberghe and Janssen [38] tissue digestion by nitric acid and boiling may have led to destruction of smaller microplastics and, hence, to an underestimation of microplastic levels in the mussels. With micro-Raman-spectrometry, no unambiguous identification of the particles as microplastic was possible. The authors assumed that this might be due to effects of the digestion procedure on the plastic matrix. Similar microplastic concentrations were reported for *M. edulis* collected at the French, Belgian and Dutch North Sea coast [122], and slightly lower levels for Mediterranean mussels (*Mytilus galloprovincialis*) sampled in the estuaries of the rivers Tagus (Portugal) and Po (Italy) and in the Ebro delta (Spain [37]). *A.* *marina* from the French and Belgian North Sea coast contained on average 1.2 microplastic particles/g tissue [122].

Plastic concentrations in the gut of small (up to 10 cm standard length) pelagic fish in the North Pacific gyre were studied by Boerger et al. [160]. Fish from six species were caught with manta trawls. Approx. 35 % of the fish had plastic particles (on average 2 items) in their stomach. Most of the ingested plastics were fragments (94 %) and had a size of 1.0–2.8 mm. Lusher et al. [161] evaluated the plastic content in the gastrointestinal tract of 5 pelagic and 5 demersal fish species caught in the English Channel. All items, which were not classified as natural prey of the fish, were analysed by FT-IR. Items consisting of plastic or the semi-synthetic fibre rayon were found in 37 % of the analysed fish (on average 1.9 items/fish). There was no significant difference in the number of ingested items between pelagic and demersal species. The ingested material mainly consisted of fibres (68 %), fragments (16 %) and beads (12 %); 92 % of the items were smaller than 5 mm, with particles of 1.0–2.0 mm size being most common. More than half of the items (58 %) were identified as rayon, 36 % as PA, 5 % as PES, 1 % as PS, 0.3 % as PE and 0.3 % as acrylic. Polymers with a lower density were predominantly detected in pelagic fish species, those with a higher density in demersal species. Microplastic particles (0.2–5 mm; due to the possibility of airborne contamination fibres were not included in the evaluation) were found in the intestinal tracts of 5 out of 7 fish species sampled in the North Sea. Overall, 2.6 % of the fish had ingested microplastics [34]. In an analysis of the microplastic content in the gastrointestinal tract of fish from five commercially used species sampled in the Adriatic Sea, microplastic items were found in 28 % of the fish (1–2 items/fish [33]).

Plastic items, including microplastics, have also been shown to be ingested by seabirds and marine mammals. For instance, plastic content in the stomach of dead, beach-washed northern fulmars (*Fulmarus glacialis*) is monitored in the context of the OSPAR ecological quality objective for marine litter stating that less than 10 % of northern fulmars should have >0.1 g plastic particles in their stomachs [162]. Fulmar stomachs were shown to contain macro- and microplastics. However, the latter were not quantified separately [163, 164]. Microplastics and small macroplastics (0.5–30 mm) were detected in the scats (faeces) of fur seals (*Arctocephalus tropicalis*, *Arctocephalus gazella*) on Macquarie Islands, between New Zealand and Antarctica [165]. Plastic particles were collected visually and analysed by SEM and FT-IR. Most scats contained 1 plastic item; particles mainly consisted of PE and had a size between 2 and 5 mm. Microplastics were also found in the digestive tract of a whale (*Mesoplodon mirus*) stranded on the Irish coast [166].

Little information is available on the uptake of microplastics by freshwater organisms in the field. In a preliminary study, Sanchez et al. [167] examined wild gudgeons (*Gobio gobio*), caught in French streams. The digestive tract of the fish was analysed visually for microplastics. Based on first results, 12 % of the gudgeons had microplastics in their intestine. The abundance of microplastics increased with the anthropogenic impact on the rivers. While no microplastics were found in the intestine of fish captured at three sites characterised by a low anthropogenic influence, the highest percentage of fish with microplastics in their intestine (approx. 20–30 %) was recorded at two sites in urban areas.

### Uptake and excretion by terrestrial organisms

Very little information is available on the uptake of microplastics by terrestrial organisms. Ugolini et al. [168] performed a laboratory study with the sand hopper *Talitrus saltator*, an amphipod inhabiting sandy coasts. In *T.* *saltator*, which had been fed with dry fish food mixed with 10 % (w/w) of PE microspheres (10–45 µm), ingestion of the microspheres was demonstrated. Within 24 h most microspheres were excreted, within a week all microspheres.

### Summary: uptake and trophic transfer

A wide variety of aquatic organisms have been shown to ingest microplastics. In most cases, particles were only detected in the intestinal tract and excreted rapidly. Translocation from the intestinal tract to the circulatory system or surrounding tissues was observed in some species for very small microplastics. In field studies, microplastics were ingested, but the ingested quantities were low. Microplastics are transferred in the food chain. Yet so far, there are no data demonstrating their bioaccumulation or biomagnification.

## Effects of microplastics on the environment

Microplastics may have very different types of effects on the environment: they may physically (mechanically) affect organisms, act as vectors for hydrophobic pollutants and as substrates for organisms, and affect sediment properties.

### Physical effects of microplastics

Macroplastics physically affect marine organisms. Especially for air-breathing animals, entanglement may result in death. The ingestion of macroplastic items may reduce the amount of consumed food and, consequently, the organisms’ fitness. Macroplastics can also block the intestinal tract and cause internal injuries [58, 75, 169–171]. It has been assumed that microplastics cause similar effects in smaller organisms, mainly with regard to the physical obstruction of feeding and digestion [6, 7, 71, 75]. Sharp-edged microplastics may injure gill tissues and the intestinal tract [7, 144]. In the following, the available data on physical effects of microplastics on aquatic organisms are summarised (see also Additional file 1: Table S3). While studies dealing exclusively with effects of nanoplastics were not considered, results of comparative studies of nano- and microplastics have been included.

#### Physical effects of microplastics on marine organisms

To date, most studies were performed with marine invertebrates. In larvae of the sea urchin *T.* *gratilla* exposed for 5 days to fluorescent PE microspheres (10–45 µm, 10^3^–3 × 10^5^ items/L), effects were only observed at the highest tested concentration. Body width was significantly lower than in the control; survival was reduced to approx. 50 % of the control level, but this effect was not significant [151].

In the marine copepod *Centropages typicus*, a 24-h exposure to concentrations ≥7 × 10^6^ items/L of fluorescent PS microspheres (7.3 µm) led to significantly reduced ingestion of algae [146]. Similarly, *Calanus helgolandicus* exposed for 24 h to PS spheres (20 µm, 7.5 × 10^4^ items/L) ingested 11 % less algae than the controls. In addition, exposed copepods preferentially ingested smaller algae. During a 6-day exposure of *C.* *helgolandicus* to the same type and concentration of PS spheres, egg production was not significantly affected, but egg size was reduced during the second half of exposure. This effect was attributed to energy depletion [172].

In 96-h acute tests, survival of adults and nauplii of the copepod *T.* *japonicus* was not affected by nano- (50 nm) and microsized (0.5 and 6 µm) PS spheres at concentrations up to 1.1 × 10^15^ (50 nm), 1.1 × 10^12^ (0.5 µm) and 6.6 × 10^8^ items/L (6 µm). Chronic effects of these three sizes of PS spheres were studied in a two-generation test with *T.* *japonicus*. The nanosized (50 nm) spheres led to a significant reduction in survival of the first (*F*_0_) and the second generation (*F*_1_) at concentrations ≥4.6 × 10^12^ items/L. In *F*_0_ and *F*_1_, the development from nauplius to copepodid was delayed at 4.6 × 10^12^ items/L (see also Additional file 1: Table S3). For both sizes of microspheres, the pattern of toxicity was different. The 0.5-µm microspheres caused an increased development time (both from nauplius to copepodite and from nauplius to adult) and a reduced survival of the *F*_1_ at the highest concentration (9.1 × 10^10^ items/L). The 6-µm microspheres, for which the highest concentration contained 5.2 × 10^7^ items/L, had no effect on survival and development of both copepod generations. However, microspheres of both sizes significantly reduced fecundity of the *F*_0_ and the *F*_1_ at all tested concentrations, i.e. the lowest observed effect concentration (LOEC) was ≤4.6 × 10^8^ items/L for the 0.5 µm spheres and ≤2.6 × 10^5^ items/L for the 6 µm spheres. The effects on fecundity may have been a consequence of a reduced quantity of ingested food associated to the presence of larger amounts of ingested microspheres [149]. It is well known that reduced growth often leads to a reduced fecundity [173–175].

In juveniles of the marine isopod *I.* *emarginata* fed for 6–7 weeks (2 moult cycles) with agar-based food containing seaweed powder and fluorescent microspheres (10 µm, approx. 12 items/mg food), PS fragments (1–100 µm, 20 fragments/mg food) or acrylic fibres (0.02–2.5 mm, 0.3 mg/g food), no significant effects on survival, growth and duration of the intermoult period were recorded [156].

Mussels (*M.* *edulis*) were exposed for 3 h to fluorescent PS microspheres (3.0 and 9.6 µm, 4.3 × 10^4^ items/L) and then transferred to control water. Effects on their feeding rate, and on haemocyte viability, phagocytic activity of the haemocytes and ability of the haemocytes to cope with oxidative stress were evaluated 3–48 days after transfer to control water. Neither the 3.0 µm nor the 9.6 µm microspheres had any significant effect on the evaluated endpoints [145]. Von Moos et al. [152] exposed *M.* *edulis* for 3–96 h to 2.5 g/L (approx. 2.7 × 10^7^ to 3.6 × 10^7^ items/L, see above) of HDPE fluff (0–80 µm). In exposed mussels, granulocytoma formation (indicating an inflammatory response) was significantly increased, and lysosomal membrane stability was significantly reduced. No effects were recorded on the condition index, the neutral lipid content and the accumulation of lipofuscin (a biomarker of oxidative stress) in the digestive tract. A 14-day exposure of *M. edulis* to PS microspheres (1.1 × 10^5^ items/L) with diameters of 10, 30 and 90 µm led to a significant increase in energy consumption, but did not significantly affect the overall energy budget of the mussels [39].

Acute effects of PE microspheres (1–5 µm) were studied in juveniles of common goby (*Pomatoschistus microps*). After 96 h exposure to two concentrations of microspheres (18.4 and 184 µg/L), acetylcholinesterase activity was significantly reduced (to approx. 80 % of the control level), while survival and other biomarkers (see Additional file 1: Table S3) were not affected [176].

In a water/sediment study, lugworms (*A.* *marina*) were exposed for 28 days to sediment containing unplasticised PVC (uPVC) granules (mean size: 130 µm; 5, 10 and 50 g uPVC/kg sediment ww). During the first 2 weeks of exposure, feeding rate of lugworms was significantly reduced at 50 g/kg sediment ww. However, during the third and fourth week of exposure, feeding rate in the controls decreased to levels close to those observed at 50 g/kg ww. No clear effect on feeding was seen at 5 and 10 g/kg ww. Phagocytic activity of coelomic fluid was significantly increased at 5 and 50, but not at 10 g/kg ww. At 10 and 50 g/kg ww, total available energy reserves were significantly lower than in the controls. Yet, weight of the uPVC exposed worms at the end of the experiment did not significantly differ from the control value. In a second experiment, *A.* *marina* was exposed for 51 h to 50 g uPVC/kg ww. The frequency of egestion events (evaluated during the last 3 h of exposure) was significantly reduced at 50 g/kg ww. In a further experiment, lugworms were exposed for 7 days to sediment with 10, 50 and 100 g silica/kg ww. Since exposure to silica did not significantly affect the number of faecal casts, the reduced organic content did not appear to be the cause for the reduced feeding activity. It was assumed that the reduced egestion frequency of the worms at 50 g uPVC/kg ww might be due to a lower feeding activity or a reduced uptake efficiency, possibly caused by reduced adhesion of uPVC particles (as compared to sediment) to the feeding apparatus of *A.* *marina* [177].

In a recent 14-days water/sediment test with *A.* *marina* exposed to PS microspheres (1.1 × 10^5^ items/kg sediment) with diameters of 10, 30 and 90 µm, protein content of the exposed lugworms was significantly increased, but the overall energy budget was not affected [39]. The shorter exposure duration and the more regular form of the microplastics in this study [39] have probably contributed to the difference between the results of the two water/sediment studies. In addition, the LOEC of 10 g/kg sediment ww obtained in the 28-days test [177] corresponds to a numerical concentration of roughly 8 × 10^5^ items/kg sediment ww (see Additional file 1: Table S3), i.e. a higher concentration than used in the 14-days test [39].

#### Physical effects of microplastics on freshwater organisms

So far, very few data are available on effects of microplastics on freshwater organisms. Rochman et al. [178, 179] performed a study with Japanese medaka (*Oryzias latipes*) to evaluate the uptake of contaminants from microplastics into fish and resultant effects (see next section). This study included a treatment with virgin microplastics (pre-production LDPE pellets ground to <500 µm). Fish were fed for 2 months at a rate of 2 % bodyweight per day with a diet containing 10 % (w/w) virgin microplastics. This diet was prepared by reducing the dextrin content of the food and, instead, adding the microplastics. Consequently, it had a lower energy density than the control diet. An appropriate negative control receiving food with the same energy density as the fish exposed to microplastics would have been desirable (see e.g. [180]) but was not included. Survival of the fish receiving the microplastics-containing diet was not affected. However, 46 % of these fish exhibited severe glycogen depletion in the liver. This effect, which was not observed in the controls, was most likely caused by the reduced energy content of the microplastics-containing food. Following exposure to microplastics, the incidence of fatty vacuolar degeneration in medaka liver was slightly increased, while gonad histology was not affected. The microplastic treatment had no significant effect on expression of *cyp1a*, vitellogenin I and oestrogen receptor α in male and female fish, and on expression of choriogenin H in male fish. In female fish, choriogenin H expression was significantly reduced after 2 months exposure, an effect interpreted by Rochman et al. [179] as early warning sign of endocrine disruption. However, in view of the lower energy density of the microplastics-containing food it appears likely that the reduced choriogenin expression is an effect of the glycogen depletion described above, i.e. should not be considered as endocrine disruption as defined in [181]. Unfortunately, Rochman et al. [178, 179] do not provide any information on effects of the microplastics treatment on fish growth.

#### Summary: physical effects of microplastics

Physical effects on marine organisms were shown to occur at high concentrations of microplastics. The observed effects appear to be mainly due to the ingestion of microplastics leading to a reduced uptake of food, which in turn results in lower energy reserves and associated effects on other physiological functions. Studies on possible toxic effects of microplastics on freshwater organisms are scarce, effects on terrestrial biota have so far not been investigated [72, 77].

### Microplastics as vectors for pollutants

#### Sorption of pollutants to microplastics

Hydrophobic organic pollutants sorb to microplastics, which are hydrophobic and have a large surface to volume ratio. There is clear evidence that contaminants such as hexachlorinated hexanes, polycyclic aromatic hydrocarbons (PAHs), polychlorinated biphenyls (PCBs) and polybrominated diphenyl ethers (PBDEs) are enriched on microplastics [11, 14, 56, 62, 71]. Sorption and desorption processes depend on the polymer type. For instance, phenanthrene reached much higher equilibrium concentrations on PE than on PP and PVC granules (200–250 µm): distribution coefficients (*K*_d_) are 38,100 L/kg for PE, 2190 L/kg for PP and 1650 L/kg for PVC. These *K*_d_ values are much higher than those for sorption of phenanthrene to two sediments (19 and 135 L/kg). However, when normalising distribution coefficients to the organic carbon content of plastics and sediments (i.e. when comparing *K*_OC_ rather than *K*_d_ values), differences between plastics and sediments are strongly reduced. The K_OC_ for sorption of phenanthrene to PE granules (44,500 L/kg) is by a factor of 2–4 higher than those for the sediments (10,400 and 20,100 L/kg), while the *K*_OC_ values for PP and PVC granules (2560 and 4340 L/kg) are lower than those derived for the sediments [182]. For PCBs, Velzeboer et al. [183] also found a similar magnitude of sorption to microplastics (PE microspheres of 10–180 µm size) and sediment organic matter.

#### Transport of pollutants sorbed to microplastics

Since microplastics can be transported over long distances, it has been proposed that they may function as vectors for sorbed hydrophobic pollutants. Such pollutants might e.g. be transported to remote sites as the Arctic [121, 182]. The relevance of marine plastics (including both micro- and macroplastics) as transport vectors for PCBs, PBDEs and perfluorooctanoic acid (PFOA) to the Arctic was evaluated by Zarfl and Matthies [121]. Based on estimated amounts of plastics and pollutants in the oceans, sorption of the pollutants to plastics, and ocean current velocities they derived a rough estimate of plastic-mediated mass fluxes of PCBs, PBDEs and PFOA. These mass fluxes were by factors of 10^3^–10^6^ lower than mass fluxes via atmospheric transport and transport with water. Therefore, it was concluded that for most substances, plastics are no relevant vectors for transport to the Arctic. Yet, plastic-mediated transport might increase the mobility of highly hydrophobic substances, which are due to their sorption to sediment quickly removed from the water column.

#### Uptake of pollutants sorbed to microplastics and resultant effects

Given that (1) concentrations of pollutants on microplastics can be several orders of magnitude higher than in the surrounding water and (2) microplastics are ingested by a wide variety of organisms, it has been postulated that microplastics may lead to an increased uptake of pollutants by aquatic organisms (see e.g. [25]). Such an uptake requires desorption of the contaminants in the organisms. Addition of the digestive surfactant sodium taurocholate was shown to enhance desorption of phenanthrene from PE, PP and PVC microplastics [182]. Similar results were obtained by Bakir et al. [184] for various organic pollutants sorbed to PE and PVC microplastics. Several studies have demonstrated that contaminants, which had been sorbed to microplastics, are transferred to organisms ingesting these microplastics. For instance, nonylphenol and phenanthrene that had been sorbed to PVC microplastics were detected in the tissue of *A.* *marina* exposed for 10 days to these microplastics [185].

Besseling et al. [155] exposed *A. marina* for 28 d to sediment contaminated with low PCB concentrations (5.28 µg PCBs/kg dw)—either alone or in combination with pre-production PS particles (400–1300 µm; 0.074, 0.74 and 7.4 % of sediment dw) previously equilibrated for 6 weeks with the sediment. In the presence of 0.074 % PS particles, PCB concentrations in *A.* *marina* were by a factor of approx. 1.1–1.5 higher than in PCB-contaminated sediment without microplastics. At 0.74 and 7.4 % PS particles, PCB concentrations in the worms were lower than with 0.074 % PS, but remained in most cases above levels in the PCB-contaminated sediment without microplastics. The authors concluded that PS microparticles had a relatively limited effect on uptake of PCBs by *A.* *marina*. Feeding activity of the lugworms decreased with increasing microplastics content. Worms in all treatments lost weight, and weight loss increased with increasing microplastics concentration. It was suggested that ingestion of the relatively large microplastic particles might have led to physical stress. In addition, organic matter content of the sediment was reduced in the presence of microplastics, i.e. the worms had to ingest larger amounts of sediment.

Rochman et al. [178, 179] performed a two-month experiment with adult medaka (*O.* *latipes*) that received control food, or food containing virgin microplastics (see previous section) or contaminated (‘marine’) microplastics. The latter were prepared by exposing pre-production LDPE pellets for 3 months at a marine site. Pellets were then ground to <500 µm and incorporated into fish food. The diets containing microplastics (10 % w/w) had a lower energy density than the control diet. At the end of exposure, levels of PAHs, PCBs and PBDEs in fish that had received marine microplastics were higher than in the control and in the virgin microplastic treatment. Marine microplastics had no significant effect on survival and expression of *cyp1a*. However, they caused more pronounced histopathological changes in the liver than virgin microplastics: 74 % of the fish exposed to marine microplastics exhibited severe glycogen depletion (virgin microplastics: 46 %), 47 % fatty vacuolar degeneration (virgin microplastics: 29 %) and 11 % single cell necrosis (virgin microplastics: 0 %). In female medaka fed with marine microplastics, the expression of vitellogenin, choriogenin H and oestrogen receptor *α* was slightly lower than in fish fed with virgin microplastics and significantly lower than in the control fish. These effects were considered as indicators of endocrine disruption [179], but are most likely related to the observed energy depletion. In this context, it is of note that reduced vitellogenin levels can only be interpreted as indicator for endocrine activity, if there is no systemic toxicity [186].

#### Relevance of microplastics as vector for pollutants

As outlined above, there is clear evidence that hydrophobic contaminants are enriched on microplastics and transferred to organisms ingesting these microplastics. However, it has been questioned, if the transport of sorbed pollutants by microplastics is a relevant factor contributing to accumulation and adverse effects in the environment, i.e. if microplastics transport pollutants in sufficiently high concentrations to biota [99, 187]. In this context, the contribution of the uptake via microplastics to the total uptake of a pollutant (including uptake via integument, gills and food) has to be considered. Since microplastics are in most cases excreted by the organisms that have ingested them, desorption rates of the pollutants from the microplastics in the intestinal tract are important. Modelling approaches have been used to assess the relative contribution of microplastics as vectors to the overall uptake of hydrophobic organic pollutants by *A.* *marina* [187, 188] and piscivorous fish [104].

Koelmans et al. [187, 188] developed a biodynamic model for PCB accumulation by *A.* *marina* in an environment containing PS and PE microparticles. Different uptake processes and desorption in the intestinal tract were considered. Bioaccumulation of various PCBs was modelled in the presence of three concentrations (0.1, 1 and 10 % of sediment dw) of microplastics (approx. 1 mm) and in the absence of microplastics. PS microparticles had no significant effect on bioaccumulation of PCBs in lugworms. For PE, the model predicted a decrease in steady-state bioaccumulation for PCBs with log K_OW_ values >5–6. When tissue concentrations increase to levels typical for substances with such high K_OW_, the gradient between concentrations in tissue and on the ingested PE microparticles may become negative. Consequently, PCBs may be resorbed to the microplastics, i.e. bioaccumulation is attenuated. Based on these results, Koelmans et al. [187] concluded that the contribution of microplastics to bioaccumulation can be assumed to be not very relevant.

Similar results were obtained by Gouin et al. [104] with two modelling approaches. Using an equilibrium partitioning model significant (>1 %) partitioning to plastics was only predicted for environments with very high plastic concentration and limited natural organic matter. Based on the steady-state food-web component of the bioaccumulation model of Arnot and Gobas [189], into which 10 % PE microplastics were included as additional component of the diet, a reduced bioaccumulation was obtained for substances with log *K*_OW_ values between 5.5 and 6.5. As outlined above, this reduction is due to the high affinity of the microplastics to the pollutants, which prevents transfer of the pollutants to the fish. Gouin et al. [104] concluded that microplastics have a limited relevance as vector for the transfer of hydrophobic pollutants to fish. A number of uncertainties were identified, which include the effects of fouling on sorption of pollutants to microplastics, gut retention times of microplastics and uptake rates of microplastics into the tissue of an organism.

### Microplastics as substrates for organisms

Many marine organisms live attached to debris [190, 191]. Due to plastics, the amount of floating debris in the oceans has greatly increased [190]. This is most relevant in the open ocean, where only very limited floating substrate is available [128, 192]. Plastic debris is often colonised by microorganisms and—depending on its size—also by larger organisms. PE microplastics and small macroplastics collected in the surface layer of the North Atlantic were colonised by a variety of organisms including bacteria, cyanobacteria, diatoms, ciliates and radiolaria [191]. Bryozoans were identified on 3–5 mm-sized microplastics sampled in the surface layer at different sites around Australia [193]. Microplastic particles are also used as oviposition sites by the sea skater *Halobates*, an insect living at the sea/air interface in the open ocean [128, 192]. The strong increase in microplastics in the North Pacific gyre, which was observed between 1972–1987 and 1999–2010, was associated with a considerable increase in the number of eggs, juveniles and adults of *Halobates sericeus*. Possible effects of the increased abundance of *H.* *sericeus* on other species have so far not been investigated [128].

Given that plastics can be transported over long distances, they may contribute to the dispersal of species [75, 190]. This includes invasive species [190] and species causing harmful algal blooms [194]. Due to their low size, microplastics may especially facilitate transport of microorganisms (including pathogens) and other very small organism. So far, it is not known if the transport of species with microplastics has any significant effect on species assemblages [158, 195].

### Effects of microplastics on sediment properties

Carson et al. [134] used artificially constructed beach sediment cores containing 1.5, 7.3, 15.9 and 29.4 % (w/w) of small plastic particles (<10 mm) to investigate effects on water permeability and heat transfer. Notably, mean size of plastic particles was higher than mean grain size of the sediment. At 15.9 and 29.4 % (w/w) plastics, water permeability was significantly increased. With increasing plastic content, sediments warmed more slowly. A 16 % decrease in heat transfer was recorded at the highest plastic concentration. Possible implications of such effects on physical properties of sediments remain to be investigated.

## Do microplastics cause environmental risks?

In the context of risk assessment, polymers are—given their high molecular weight—generally considered as being of low concern. It is of note that, for this reason, registration and evaluation of polymers under REACH is usually not required unless triggered by certain additives [15]. As a first estimate of possible concern for the environment, the highest measured levels of microplastics in the environment, which were identified based on Hidalgo-Ruz et al. [11] and selected recent publications (Additional file 1: Table S1), are compared to the concentrations of microplastics, which were shown to cause physical effects in laboratory tests. In the surface layer and the water column of the oceans, maximum concentrations of 9 [11] and 10 items/L [18], respectively, were found. These concentrations are by a factor of approx. 10^4^ lower than the acute LOEC of 3 × 10^5^ items/L [151] and the chronic LOEC of ≤2.6 × 10^5^ items/L [149] obtained for marine invertebrates exposed via the water phase. Note that in the chronic test [149] a clear effect was observed at the lowest tested concentration (Additional file 1: Table S3), i.e. significant effects may be caused at a lower microplastic level. The highest microplastic concentrations measured in subtidal sediments, 2175 items/kg dw in the lagoon of Venice [22] and 3600 items/kg dw in the Rhine estuary [89], are lower than the LOEC of 10 g/kg sediment ww (1 % w/w [177]) derived in a water/sediment test with marine polychaetes, which corresponds to a numerical concentration of roughly 10^6^ items/kg sediment dw (Additional file 1: Table S3). In beach sediments, maximum levels of 109 g microplastic items/L (on the Canary Island Lanzarote [134]) and 30 % (w/w) small plastic particles (in the upper layer of Kamilo beach, Hawaii [133]) were determined. These values are higher than the LOEC obtained in the water/sediment test mentioned above (no toxicity data for organisms inhabiting beaches are available).

It should be noted that to date only relatively few studies are available on the effects of microplastics in marine organisms and even fewer on those in freshwater organisms. In several cases, only single concentrations were tested and threshold concentrations, below which no significant effects are observed in the respective test organisms, were not determined (Additional file 1: Table S3). Effects on terrestrial organisms have so far not been studied at all. Furthermore, microplastics are a very heterogeneous group of particles differing e.g. in size, shape, chemical composition and specific density. Little is known on the influence of these factors on the effects of microplastics [196].

In addition, there are a number of knowledge gaps concerning fate and occurrence of microplastics in the environment. Little information is available on fragmentation and degradation rates of macro- and microplastics. Due to methodological limitations, size distributions in the environment are only partly known, especially with regard to the smallest microplastics and nanoplastics [8, 11, 99, 196]. While there are many studies on microplastic abundance in the marine environment, data on the occurrence of microplastics in freshwater systems are limited. Information on possible hotspots and sinks is missing [97]. For the terrestrial environment, there are nearly no data on the occurrence of microplastics. These data gaps need to be filled prior to being able to perform a comprehensive assessment of possible environmental risks caused by microplastics.

Moreover, a number of possible effects of microplastics on the environment are not covered by current environmental risk assessment procedures, which have been developed for chemical substances. These include potential effects on sediment properties, and the function of microplastics as vector for the transport of pollutants, invasive species or pathogens. Assessment factors, which have been derived for the environmental risk assessment of chemicals, may not be appropriate for microplastics. For these reasons, assessing possible environmental risks caused by microplastics is not straightforward. An approach that enables a comprehensive assessment of possible environmental risks caused by microplastics remains to be developed [14, 97]. As suggested by Syberg et al. [196], such an approach should build on frameworks, which have been developed for assessing environmental risks of nanomaterials and mixtures.

It should be pointed out that based on available information microplastics are extremely persistent. For this reason and due to the fragmentation of macroplastic debris, which is abundant in the environment, concentrations of microplastics in the environment will increase at least as long as the release of plastics to the environment is not stopped [8, 25, 99]. In this context, the high concentrations in coastal sediments, which have been recorded at some sites [133, 134], are of specific concern.

## Conclusions

As a result of the widespread use of plastics there are a great number of sources of primary and secondary microplastics in the environment. First estimates indicate that the contribution of personal care products to the overall amount of microplastics in the environment is of minor relevance. Abrasion and fragmentation of larger plastic items and of materials containing synthetic polymers have been considered as much more relevant.

Based on the evaluated data, the lowest concentrations eliciting adverse effects in aquatic organisms exposed via the water are by a factor of approx. 10^4^ higher than maximum microplastic concentrations found in marine waters. The effect concentration in a water/sediment test with lugworms is higher than microplastic levels measured in subtidal sediments but in the same range as highest levels recorded in beach sediments.

Before we are able to perform a comprehensive assessment of possible environmental risks caused by microplastics, a number of data gaps (e.g. fate and effects in freshwater and soil, and size distributions in all environmental compartments) need to be filled and available environmental risk assessment procedures have to be adapted. However, given that (1) very high concentrations of microplastics have already been observed at some sites (especially in sediments and on beaches), (2) plastics are extremely persistent in the environment, (3) microplastics in the environment originate from a multitude of sources and (4) the abundance of microplastic is expected to further increase due to fragmentation of the macroplastic present in the environment, strategies should be developed to address the issue of nano-, micro- and macroplastics in the environment on a broad and global basis in order to avoid exceeding critical environmental threshold concentrations.

## Additional file

10.1186/s12302-015-0069-y Overview of ranges and mean or median values (underlined) of concentrations of microplastics (or, where specified, small plastic particles) in the marine environment based on Hidalgo-Ruz et al. [11] and selected recent publication. **Table S2.** Overview of ranges and mean or median values (underlined) of concentrations of microplastics (or, where specified, small plastic particles) in the freshwater environment. **Table S3.** Overview of effect concentrations derived in ecotoxicity tests with aquatic organisms exposed to microplastics.
