# Supplementary material for: Microplastics in the aquatic and terrestrial environment: sources (with a specific focus on personal care products), fate and effects
Source: Environ Sci Eur. 2016 Jan 6;28(1):2. doi: 10.1186/s12302-015-0069-y (PMC5044952; doi:10.1186/s12302-015-0069-y)
Supplement: Supplementary file 1 — 10.1186/s12302-015-0069-y Overview of ranges and mean or median values (underlined) of concentrations of microplastics (or, where specified, small plastic particles) in the marine environment based on Hidalgo-Ruz et al. [11] and selected recent publication. Table S2. Overview of ranges and mean or median values (underlined) of concentrations of microplastics (or, where specified, small plastic particles) in the freshwater environment. Table S3. Overview of effect concentrations derived in ecotoxicity tests with aquatic organisms exposed to microplastics. [file 12302_2015_69_MOESM1_ESM.docx]

**Supplemental data:**

**Microplastics in the aquatic and terrestrial environment:
sources (with a specific focus on personal care products), fate and effects**

K. Duis and A. Coors

**Table S1**. Overview of ranges and mean or median values (underlined) of concentrations of microplastics (or, where specified, small plastic particles) in the marine environment based on Hidalgo-Ruz et al. (2012) and selected recent publication.

**Table S2**. Overview of ranges and mean or median values (underlined) of concentrations of microplastics (or, where specified, small plastic particles) in the freshwater environment.

**Table S3**. Overview of effect concentrations derived in ecotoxicity tests with aquatic organisms exposed to microplastics.

Table S1. Overview of ranges and mean or median values (underlined) of concentrations of microplastics (or, where specified, small plastic particles) in the marine environment based on Hidalgo-Ruz et al. (2012) and selected recent publication.

| **Compartment, sampling site** | **Numerical concentration** | | **Mass concentration** | **Size range of microplastics** | **Used method** | **Reference** |
| --- | --- | --- | --- | --- | --- | --- |
|  | **per area** | **per volume or weight** |  |  |  |  |
|  | | | | | | |
| **Sea surface layer** | | | | | | |
| Various sites^a^ | 0.00008–5 items/m^2^ | 0.022–8,654 items/m^3^ | – | Differ between studies^a, b^ | | Hidalgo-Ruz et al. [11] |
| North Pacific (including North Pacific gyre) | Overall range: 0.0–12.3 items/m^2^ Median in North Pacific gyre: 0.03 items/m^2^ Median outside North Pacific gyre: 0.0 items/m^2^ | – | – | Plastic items were typically mm sized, exact size limits not indicated | Sampling by neuston net (335 µm), visual collection and identification | Law et al. [20] |
| North Pacific gyre | – | 0.092 ^c^–32.8 items/m^3^ Median: 0.4 items/m^3^ | – | Differ between studies^d^ | | Goldstein et al. [128] |
| Alaska | – | 0.0–0.4 items/m^3^ Median: 0.0 items/m^3^ | – |  |  |  |
| California Current | – | 0.0–3.1 items/m^3^ Median: 0.01 items/m^3^ | – |  |  |  |
| Eastern Tropical Pacific | – | 0.0–0.04 items/m^3^ Median: 0.01 items/m^3^ | – |  |  |  |
| Mediterranean Sea | 0–0.892 items/m^2^ Mean: 0.116 items/m^2^ | – | 0–2.28 mg/m^2^ Mean: 0.20 mg/m^2^ | 0.3–5 mm | Sampling by manta trawl (333 µm), density separation (no information provided on type of used solution), visual identification with a binocular microscope | Collignon et al. [126] |
|  | 0–0.69 items/m^2^ Mean: 0.062 items/m^2^ | – | – | Small plastic items: 0.2–10 mm | Sampling with a wp2 net (0.2 mm), density separation (no information provided on type of used solution), visual identification with a binocular microscope | Collignon et al. [S1] |
|  | Mean: 0.13 items/m^2^ | – | Mean: 0.06 mg/m^2^ | 0.33–5 mm | Sampling by manta trawl (333 µm), sieving (300 µm, 5 mm), visual sorting and identification (stereo microscope) | Faure et al. [S2] |
| **Compartment, sampling site** | **Numerical concentration** | | **Mass concentration** | **Size range of microplastics** | **Used method** | **Reference** |
|  | **per area** | **per volume or weight** |  |  |  |  |
|  | | | | | | |
| **Sea surface microlayer** | | | | | | |
| Nearshore and offshore Goeje Island, southern Korea | – | Paint particles: Mean: 195 items/L Other microplastics: Mean: 16 items/L | – | 0.75 µm–approx. 5 mm (larger microplastics were not sampled effectively) | Sampling of top 1 mm of water using a 2 mm sieve lowered to touch the water surface (particles were trapped by surface tension), filtration (0.75 µm), identification by Fourier transform infrared spectroscopy (FT-IR) | Song et al. [21] |
| **Water column** | | | | | | |
| Various sites^a^ | – | 0.014–12.5 items/m^3^ | – | Differ between studies^a, b^ | | Hidalgo-Ruz et al. [11] |
| Northeastern Pacific and coastal British Columbia | – | 8–9,810 items/m^3^ Mean: 2,080 items/m^3^ | – | 63 µm–5 mm | Bulk sampling at 4.5 m depth with the ship’s saltwater intake system, sieving (lowest pore size: 62.5 µm), digestion with concentrated HCl at 80–90°C, filtration (0.45 µm), microscopic identification | Desforges et al. [18] |
| Yangtze estuary | – | Mean: 4,137 items/m^3^ | – | 0.5 mm–approx. 5 mm | Sampling at 1 m depth with a pump, sieving (32 µm), digestion with 30% H_2_O_2_ (for samples containing large quantities of organic matter), density separation (saturated zinc chloride solution), filtration (1.2 µm), microscopic identification | Zhao et al. [131] |

| **Compartment, sampling site** | **Numerical concentration** | | **Mass concentration** | | **Size range of microplastics** | **Used method** | **Reference** |
| --- | --- | --- | --- | --- | --- | --- | --- |
|  | **per area** | **per volume or weight** |  |  |  |  |  |
|  | | | | | | | |
| **Coastal sediments (beaches)** | | | | | | | |
| Various sites^a^ | 0.21– >77,000 items/m^2^ | – | – | Differ between studies^a, b^ | | | Hidalgo-Ruz et al. [11] |
| Belgian beaches | – | Mean: 93 items/kg dw | – | 38 µm–1 mm | | Bulk sampling at high tide line, in the middle of the intertidal area and in subtidal zone, density separation (concentrated NaCl), sieving (38 µm), visual analysis (stereo microscope) with quality control (extraction of particles from sediments spiked with known microplastic levels, obtained recovery rates used as correction factors when deriving microplastic concentrations for the beach samples), identification by FT-IR | Claessens et al. [110] |
| Belgian beaches | – | 2–48 items/kg dw Mean: 13 items/kg dw | – | Lower size limit not indicated^e^, upper size limit: 1 mm | | Bulk sampling (upper 5 cm) at the high and low tide line, elutriation, sieving (35 µm), extraction with NaI (density: 1.6 g/cm^3^), filtration (5 µm), visual identification | Van Cauwen­berghe et al. [105] |
| Beach on the North Sea island Norderney, Germany | – | 1–4 items/kg dw excluding fibres  (due to considerable number of fibres in procedural controls)  Mean values at three studied sites: 1.3, 1.7 and 2.3 items/kg dw | – | Lower size limit: not indicated, upper size limit: 1 mm | | Bulk sampling (upper 3 cm) at the high and low tide line, sieving (1 mm), two-step air-induced overflow extraction, (1) concentrated NaCl solution (1.2 g/cm^3^), (2) NaI solution (1.8 g/cm^3^), visual identification (stereo microscope), analysis of a subsample by thermal desorption pyrolysis GC/MS | Dekiff et al. [49] |

| **Compartment, sampling site** | **Numerical concentration** | | **Mass concentration** | | **Size range of microplastics** | **Used method** | **Reference** |
| --- | --- | --- | --- | --- | --- | --- | --- |
|  | **per area** | **per volume or weight** |  |  |  |  |  |
|  | | | | | | | |
| Chilean continental coast | Mean: 27 items/m^2^ | – | – | Small plastic particles:  1–10 mm | | Collection of the upper 2 cm of the beach surface at the high tide line, sieving (1 mm), visual sorting and identification | Hidalgo-Ruz & Thiel [101] |
| Easter Islands coast | Mean: 805 items/m^2^ | – | – |  |  |  |  |
| South Korean beaches, close to estuary of Nakdong River | Mean values: 8,205 items/m^2^ before rainy season,  27,606 items/m^2^ after rainy season | – | – | 1–5 mm | | Sampling in areas with highest and lowest amount of beach debris based on visual assessment, sieving of the upper 5 cm of the sediment (5 mm and 1 mm mesh width), visual sorting and identification | Lee et al. [102] |
| Beaches on the Canary Island Fuerteventura | – | – | 1–30 g/L  sediment | Lower size limit: not indicated, upper size limit: at least 2 dimensions <5 mm | | Bulk sampling (upper ≤1 cm) around the high tide line, density separation in seawater, visual collection and identification | Baztan et al. [133] |
| Beaches on the Canary Island Lanzarote | – | – | <1–109 g/L sediment |  |  |  |  |
| Beaches on the Canary Island La Graciosa | – | – | <1–90 g/L sediment |  |  |  |  |
| Kamilo beach, southern Hawaii | – | – | In upper 5 cm:  Mean: 3.3% (w/w), max.: 30% (w/w) | Small plastic particles Lower size limit: not indicated,  upper size limit <10 mm | | Bulk sampling (5 cm diameter sediment cores) at, below and above the high tide line, density separation in concentrated NaCl solution (1.2 g/cm^3^), visual identification, analysis of a subsample by FT-IR | Carson et al. [134] |
| Waikapuna beach, southern Hawaii | – | – | In upper 5 cm:  Mean: 0.1% (w/w), max.: 0.8% (w/w) |  |  |  |  |

| **Compartment, sampling site** | **Numerical concentration** | | **Mass concentration** | **Size range of microplastics** | **Used method** | **Reference** |
| --- | --- | --- | --- | --- | --- | --- |
|  | **per area** | **per volume or weight** |  |  |  |  |
|  | | | | | | |
| **Subtidal sediments** | | | | | | |
| Various sites^a^ | – | approx. 18–125 items/L | – | Differ between studies^a, b^ | | Hidalgo-Ruz et al. [11] |
| Harbour sediment, Belgian coast | – | Mean value:  167 items/ kg dw | – | 38 µm–1 mm | Bulk sampling (Van Veen grab), density separation (concentrated NaCl), sieving (38 µm), visual analysis (stereo microscope) with quality control (extraction of particles from sediments spiked with known microplastic levels, obtained recovery rates used as correction factors when deriving microplastic concentrations for the beach samples), identification by FT-IR | Claessens et al. [110] |
| Sedimentation zone of Scheldt river, Belgian coast | – | Mean value:  92 items/kg dw | – |  |  |  |
| Belgian coast, 21 km offshore | – | Mean value: 105 items/kg dw | – |  |  |  |
| Dutch North Sea coast | – | 100–720 items/kg dw Mean: 440 items/kg dw | – | 1 µm–5 mm | Bulk sampling (grab samples) of surface sediments, density separation with NaCl solution (1.2 g/cm^3^), filtration (0.7 µm), microscopic analysis | Leslie et al. [89] |
| Dutch Wadden Sea | – | 770 items/kg dw | – |  |  |  |
| Rhine estuary | – | Mean: 3,300 items/kg dw  (mean of 2 values: 3,010 and 3,600 items/kg dw) | – |  |  |  |
| Lagoon of Venice, Italy (approx. 1 m water depth) | – | 672–2,175 items/kg dw | – | 32 µm–1 mm | Bulk sampling (box corer) of the upper 5 cm, density separation with concentrated NaCl (1.2 g/cm^3^), sieving (32 µm), collection on filters (0.7 µm), identification by micro-FT-IR | Vianello et al. [22] |

| **Compartment, sampling site** | **Numerical concentration** | | **Mass concentration** | **Size range of microplastics** | **Used method** | **Reference** |
| --- | --- | --- | --- | --- | --- | --- |
|  | **per area** | **per volume or weight** |  |  |  |  |
|  | | | | | | |
| North Sea: former sewage sludge disposal site | – | Approx. 16 items/L sediment ^f^ | – | Lower size limit: not indicated, upper size limit: 1 mm | Bulk sampling (van Veen grab) of the upper 5–10 cm, density separation with saturated NaCl solution, identification with FT-IR | Browne et al. [81] |
| North Sea: reference site | – | Approx. 4 items/L sediment ^f^ | – |  |  |  |
| English Channel: former sewage sludge disposal site | – | Approx. 20 items/L sediment ^f^ | – |  |  |  |
| English Channel: reference site | – | Approx. 8 items/L sediment ^f^ | – |  |  |  |
| **Deep sea sediments** | | | | | | |
| Northeast Atlantic Ocean, southwest Indian Ocean, Mediterranean Sea (300–3,500 m water depth) | – | 28–800 items/L sediment  (exclusively fibres) Mean: 268 items/L sediment | – | Lower size limit: 1.6 µm or 32 µm^g^; upper size limit not indicated  (most microfibers had a length of 2–3 mm and a diameter <0.1 mm) | Bulk sampling of the upper 1–5 cm with megacorers, box corers or push-corers; density separation using concentrated NaCl solution (1.2 g/cm^3^) or an adapted Ludox-TM 40 method (1.2 g/cm^3^; Griffith et al. 1990), sieving (32 µm) or filtration (1.6 µm), microscopic sorting, identification with FT-IR | Woodall et al. [123] |
| Kuril-Kamchatka Trench, northwest Pacific (4,869–5,766 m water depth) | 60–2020 items/m^2^ | – | – | 300 µm–1 mm | Bulk sampling (box corer) of the upper 20 cm, sieving (1000, 500 and 300 µm), visual sorting (stereo­microscope) and identification | Fischer et al. [124] |

^a^ In this review, quantitative data from 60 studies were evaluated.

^b^ Although Hidalgo-Ruz et al. [11] have suggested a size limit of 5 mm for microplastics, some of the reviewed studies cover a wider size range. Overall, the size of the identified plastics items ranged from 1 µm to 20 mm, i.e. some studies have included small macroplastics. However, in most of the studies only items with a size <5 mm were included.

^c^ 5^th^ Percentile (the minimum is not indicated)

^d^ Data for the period from 1999 to 2010 as compiled by Goldstein et al.[128]. Data from Day and Shaw [S4], the Algalita Marine Research Foundation [S5], Gilfillan et al. [23], Doyle et al. [S6] and Goldstein et al. [128].

^e^ Following elutriation, supernatant was sieved (35 µm). However, particles in this size range are likely to have been missed by visual analysis.

^f^ Reading from a graph.

^g^ Two different methods were used to extract the microplastics.

Table S2. Overview of ranges and mean or median values (underlined) of concentrations of microplastics (or, where specified, small plastic particles) in the freshwater environment.

| **Compartment, sampling site** | **Numerical concentration** | | **Mass concentration** | **Size range of microplastics** | **Used method** | **Reference** |
| --- | --- | --- | --- | --- | --- | --- |
|  | **per area** | **per volume or weight** |  |  |  |  |
| **Streams: surface water layer** | | | | | | |
| River Danube between Vienna (Austria) and Bratislava (Czech Republic) | – | 2010:  Mean: 0.938 items/m^3^ 2012:  Mean: 0.055 items/m^3^ | 2010:  Mean: 0.011 g/m^3^ 2012:  Mean: 0.002 g/m^3^ | Small plastic items: 0.5–20 mm | Sampling of the upper 0.5 m of the water column with driftnets (500 µm), visual collection and identification | Lechner et al. [68] |
| River Rhône  (Chancy, Switzerland) | – | Mean: 0.29 items/m^3^ Median: 0.25 items/m^3^ | Mean: 0.35 mg/m^3^ Median: 0.32 mg/m^3^ | 0.3–5 mm | Sampling by manta trawl (300 µm), followed by (a) for items >1mm: visual analysis and analysis of a subsample by FT-IR; (b) for items ≤1 mm: digestion with H_2_O_2_ and visual analysis (stereo microscope) | de Alencastro [138] |
| North Shore Channel, Chicago (USA) | – | Upstream of WWTP: Mean: 1.94 items/m^3^  Downstream of WWTP: Mean: 17.93 items/m^3^ | – | 0.33–2 mm | Sampling by neuston net (333 µm), sieving (2 mm, 330 µm), digestion with 30% H_2_O_2_ and Fe (II) at 75°C, density separation (NaCl), filtration, visual analysis (dissecting microscope). Procedural controls to verify background contamination. Fibre content in procedural controls was subtracted from values counted in samples. | McCormick et al. [139] |

| **Compartment, sampling site** | **Numerical concentration** | | **Mass concentration** | **Size range of microplastics** | | **Used method** | **Reference** |
| --- | --- | --- | --- | --- | --- | --- | --- |
|  | **per area** | **per volume or weight** |  |  |  |  |  |
| **Streams: coastal sediments** | | | | | | | |
| River Rhine (Germany) | – | 228–3,763 items/kg dw | 21.8–932 mg/kg dw | 63 µm – 5 mm | | Bulk sampling of upper 2-3 cm of sediment between water line and lowest flotsam line with steel spoon (3–4 kg/sample), sieving (63, 200, 630 µm), removal of items >5 mm, density separation ( saturated NaCl), filtration, digestion with mixture of 30% H_2_O_2_ and concentrated sulfuric acid, visual sorting with naked eye (>630 µm) or binocular microscope (63-630 µm), FT-IR analysis of a subsample (630-5000 µm). | Klein et al. [112] |
| River Main (Germany) | – | 786–1,368 items/kg dw | 43.5–459 mg/kg dw |  |  |  |  |
| **Streams: submerged sediments** | | | | | | | |
| St. Lawrence River (Canada) | 0–136,926 items/m^2^  Mean: 13,759 items/m^2^ Median: 52 items/m^2 a^ | 0–1,369 items/L sediment | – | Approx. 0.5–2.5 mm | | Bulk sampling (Ponar or Peterson grab) of the upper 10–15 cm of the sediment, sieving (0.5 mm), visual sorting and identification (dissecting microscope) | Castañeda et al. [140] |
| **Lakes: surface water layer** | | | | | | | |
| Lake Geneva (Switzerland) | 0.048 items/m^2^ (single sample) | – | – | | 0.3–5 mm | Sampling by manta trawl (300 µm), sieving (5 mm), visual collection and identification (stereo microscope). Due to the presence of large amounts of pollen, only one sample was evaluated. | Faure et al. [141] |
| Lake Geneva, Lake Constance, Lake Neuchâtel, Lake Maggiore, Lake Zurich, Lake Brienz (Switzerland) | Mean: 0.091 items/m^2^ Median: 0.048 items/m^2^ | – | Mean: 0.026 mg/m^2^ Median: 0.009 mg/m^2^ | | 0.3–5 mm | Sampling by manta trawl (300 µm), followed by (a) for items >1mm: visual analysis and analysis of a subsample by FT-IR; (b) for items ≤1 mm: digestion with H_2_O_2_ and visual analysis (stereo microscope) | de Alencastro [138] |

| **Compartment, sampling site** | **Numerical concentration** | | | **Mass concentration** | **Size range of microplastics** | | **Used method** | **Reference** |
| --- | --- | --- | --- | --- | --- | --- | --- | --- |
|  | **per area** | **per volume or weight** | |  |  |  |  |  |
| Lake Superior, Lake Huron and Lake Erie (Great Lakes) | 0–0.463 items/m^2^ Mean: 0.043 items/m^2^ | – | | – | | 0.355 mm to approx. 5 mm | Sampling by manta trawl (333 µm), sieving (355 µm, 1 mm, 4.75 mm) and visual collection and identification, analysis of all particles <1 mm by scanning electron microscopy (SEM) and energy dispersive X-ray spectroscopy (EDS) | Eriksen et al. [41] |
| Lake Hovsgol (Mongolia) | 0.001–0.044 items/m^2^ Mean: 0.0203 items/m^2^ | – | | – | | 0.355 mm to approx. 5 mm | Sampling by manta trawl (333 µm), sieving (355 µm, 1 mm, 4.75 mm) digestion with 30% H_2_O_2_ and Fe (II), density separation with saltwater (1.62 g/cm^3^), microscopic identification | Free et al. [103] |
| **Lakes: beaches** | | | | | | | | |
| Beaches of Lake Garda (Italy) | Northern shore: 1,108 items/m^2^ Southern shore: 108 items/m^2^ | | – | – | | <5 mm | Bulk sampling (grid samples) of the upper 5 cm, density separation with ZnCl_2_ solution (1.6–1.7 g/cm^3^), filtration (0.3 µm), analysis by Raman microspectrometry, evaluation of a subsample by SEM | Imhof et al. [142] |
| Beaches of Lake Geneva, Lake Constance, Lake Neuchâtel, Lake Maggiore, Lake Zurich, Lake Brienz (Switzerland) | 20–7,200 items/m^2^ Mean: 1,300 items/m^2^ Median: 270 items/m^2^ | | – | 1–6,000 mg/m^2^ Mean: 920 mg/m^2^ Median: 110 mg/m^2^ | | 0.3–5 mm | Bulk sampling (grid samples) of the upper 5 cm at high tide line, density separation with NaCl (1.2 g/cm^3^), filtration followed by (a) for items >1mm: visual analysis and analysis of a subsample by FT-IR; (b) for items <1 mm: digestion with H_2_O_2_ and visual analysis (stereo microscope) | de Alencastro [138] |

^a^ Concentrations at 9 out of 10 sampling sites ranged from 0 to 243 items/m^2^.

**Table S3.** Overview of effect concentrations derived in ecotoxicity tests with aquatic organisms exposed to microplastics^a, b^

| **Test species** | **Test method (duration)** | **Particle type, size** | **Particle concentrations** | | **Endpoint** | **Effect concentration** | | **Remark** | **Reference** |
| --- | --- | --- | --- | --- | --- | --- | --- | --- | --- |
|  |  |  | **Numerical conc.** | **Mass conc.** |  |  |  |  |  |
| **Studies with marine organisms: water-only systems** | | | | | | | | | |
| **Sea urchins** | | | | | | | | | |
| *Tripneustes gratilla* | Acute (5 d) test with larvae, starting 5–8 d post fertilisation | Fluorescent polyethylene microspheres, 10–45 µm | 10^3^, 10^4^, 10^5^ and 3 x 10^5^ items/L | n.i. | Survival | LOEC | >3 x 10^5^ items/L | At 3 x 10^5^ items/L, survival clearly but not significantly reduced | Kaposi et al. [151] |
|  |  |  |  |  | Body width | LOEC | 3 x 10^5^ items/L | – |  |
|  |  |  |  |  | Post-oral arm length | LOEC | >3 x 10^5^ items/L | – |  |
| **Copepods** |  |  |  |  |  |  |  |  |  |
| *Centropages typicus* | Evaluation of acute (24 h) effect on ingestion of algae | Fluorescent polystyrene microspheres, 7.3 µm | 4 x 10^6^, 7 x 10^6^, 1.1 x 10^7^ and 2.5 x 10^7^ items/L | n.i. | Algal ingestion rate | LOEC | 7 x 10^6^ items/L | – | Cole et al. [146] |
| *Calanus helgolandicus* | Evaluation of acute (24 h) effect on ingestion of algae | Polystyrene microspheres, 20 µm | 7.5 x 10^4^ items/L | n.i. | Algal ingestion rate | Reduced by 11% based on number of ingested algae. The exposed copepods ingested smaller algae. | | – | Cole et al. [172] |
| *C. helgolandicus* | Reproduction test (3 d pre-exposure, 6 d exposure) | Polystyrene microspheres, 20 µm | 7.5 x 10^4^ items/L | n.i. | Egg production | No significant effect | | – | Cole et al. [172] |
|  |  |  |  |  | Egg size | Significant reduction during last half of exposure | |  |  |
|  |  |  |  |  | Hatching success | Reduced on d 3 of exposure, no significant effect on exposure day 1 and 6 | |  |  |

| **Test species** | **Test method (duration)** | **Particle type, size** | **Particle concentrations** | | **Endpoint** | **Effect concentration** | | **Remark** | **Reference** |
| --- | --- | --- | --- | --- | --- | --- | --- | --- | --- |
|  |  |  | **Numerical conc.** | **Mass conc.** |  |  |  |  |  |
| *Tigriopus japonicus* | Acute tests (96 h) with (a) adult females and (b) <24 h-old nauplii | Polystyrene nanospheres, 50 nm | 2.2 x 10^13^–1.1 x 10^15^ items/L | 6, 13, 31, 63, 187, 250 and 313 mg/L | Survival | LC_50_ | >1.1 x 10^15^ items/L (313 mg/L) | No mortality in both, the tests with adult females and the tests with nauplii | Lee et al. [149] |
|  |  | Polystyrene microspheres, 0.5 µm | 2.2 x 10^10^–1.1 x 10^12^ items/L |  | Survival | LC_50_ | >1.1 x 10^12^ items/L (313 mg/L) |  |  |
|  |  | Polystyrene microspheres,  6 µm | 1.3 x 10^7^–6.6 x 10^8^ items/L |  | Survival | LC_50_ | >6.6 x 10^8^ items/L (313 mg/L) |  |  |
| *T. japonicus* | Two-generation test starting with <24 h-old nauplii | Polystyrene nanospheres, 50 nm | 4.6 x 10^11^, 4.6 x 10^12^, 4.6 x 10^13,^ 9.1 x 10^13^ items/L | 0.125,1.25, 12.5 and 25 mg beads/L | **F_0_** survival | LOEC | 4.6 x 10^12^ items/L(1.25 mg/L) | Numerical concentrations were derived based on information provided by Lee et al. [149] for the acute test | Lee et al. [149] |
|  |  |  |  |  | **F_0_** development nauplius to copepodite | LOEC | 4.6 x 10^12^ items/L (1.25 mg/L) |  |  |
|  |  |  |  |  | **F_0_** development nauplius to adult | No effect at ≤4.6 x 10^12^ items/L, 100% mortality at higher concentrations | |  |  |
|  |  |  |  |  | **F_0_** sex ratio |  |  |  |  |
|  |  |  |  |  | **F_0_** fecundity (nauplii/ female, 1^st^ brood) |  |  |  |  |
|  |  |  |  |  | **F_1_** survival | LOEC | 4.6 x 10^12^ items/L (1.25 mg/L) |  |  |
|  |  |  |  |  | **F_1_** development nauplius to copepodid | LOEC | 4.6 x 10^12^ items/L (1.25 mg/L) |  |  |
|  |  |  |  |  | **F_1_** development nauplius to adult | No effect at 4.6 x 10^11^ items/L, 100% mortality at higher concentrations | |  |  |
|  |  |  |  |  | **F_1_** sex ratio |  |  |  |  |
|  |  |  |  |  | **F_1_** fecundity (nauplii/ female, 1^st^ brood) |  |  |  |  |

| **Test species** | **Test method (duration)** | **Particle type, size** | **Particle concentrations** | | **Endpoint** | **Effect concentration** | | **Remark** | **Reference** |
| --- | --- | --- | --- | --- | --- | --- | --- | --- | --- |
|  |  |  | **Numerical conc.** | **Mass conc.** |  |  |  |  |  |
| *T. japonicus* | Two-generation test starting with <24 h-old nauplii | Polystyrene microspheres, 0.5 µm | 4.6 x 10^8^ 4.6 x 10^9^, 4.6 x 10^10^,  9.1 x 10^10^ items/L | 0.125, 1.25,  12.5,  25 mg beads/L | **F_0_** survival | LOEC | >9.1 x 10^10^ items/L (>25 mg/L) | Numerical concentrations were derived based on information provided by Lee et al. [149] for the acute test | Lee et al. [149] |
|  |  |  |  |  | **F_0_** development nauplius to copepodite | LOEC | >9.1 x 10^10^ items/L (>25 mg/L) |  |  |
|  |  |  |  |  | **F_0_** development nauplius to adult | LOEC | >9.1 x 10^10^ items/L (>25 mg/L) |  |  |
|  |  |  |  |  | **F_0_** sex ratio | LOEC | >9.1 x 10^10^ items/L (>25 mg/L) |  |  |
|  |  |  |  |  | **F_0_** fecundity (nauplii/ female, 1^st^ brood) | LOEC | ≤4.6 x 10^8^ items/L (≤0.125 mg/L)^c^ |  |  |
|  |  |  |  |  | **F_1_** survival | LOEC | 9.1 x 10^10^ items/L (25 mg/L) |  |  |
|  |  |  |  |  | **F_1_** development nauplius to copepodid | LOEC | 9.1 x 10^10^ items/L (25 mg/L) |  |  |
|  |  |  |  |  | **F_1_** development nauplius to adult | LOEC | 9.1 x 10^10^ items/L (25 mg/L) |  |  |
|  |  |  |  |  | **F_1_** sex ratio | LOEC | >9.1 x 10^10^ items/L (>25 mg/L) |  |  |
|  |  |  |  |  | **F_1_** fecundity (nauplii/ female, 1^st^ brood) | LOEC | ≤4.6 x 10^8^ items/L (≤0.125 mg/L)^c^ |  |  |

| **Test species** | **Test method (duration)** | **Particle type, size** | **Particle concentrations** | | **Endpoint** | **Effect concentration** | | **Remark** | **Reference** |
| --- | --- | --- | --- | --- | --- | --- | --- | --- | --- |
|  |  |  | **Numerical conc.** | **Mass conc.** |  |  |  |  |  |
| *T. japonicus* | Two-generation test starting with <24 h-old nauplii | Polystyrene microspheres,  6 µm | 2.6 x 10^5^ 2.6 x 10^6^, 2.6 x 10^7^,  5.2 x 10^7^ items/L | 0.125, 1.25,  12.5,  25 mg beads/L | **F_0_** survival | LOEC | >5.2 x 10^7^ items/L (>25 mg/L) | Numerical concentrations were derived based on information provided by Lee et al. [149] for the acute test | Lee et al. [149] |
|  |  |  |  |  | **F_0_** development nauplius to copepodite | LOEC | >5.2 x 10^7^ items/L (>25 mg/L) |  |  |
|  |  |  |  |  | **F_0_** development nauplius to adult | LOEC | >5.2 x 10^7^ items/L (>25 mg/L) |  |  |
|  |  |  |  |  | **F_0_** sex ratio | LOEC | >5.2 x 10^7^ items/L (>25 mg/L) |  |  |
|  |  |  |  |  | **F_0_** fecundity (nauplii/ female, 1^st^ brood) | LOEC | ≤2.6 x 10^5^ items/L (≤0.125 mg/L)^c^ |  |  |
|  |  |  |  |  | **F_1_** survival | LOEC | >5.2 x 10^7^ items/L (>25 mg/L) |  |  |
|  |  |  |  |  | **F_1_** development nauplius to copepodid | LOEC | >5.2 x 10^7^ items/L (>25 mg/L) |  |  |
|  |  |  |  |  | **F_1_** development nauplius to adult | LOEC | >5.2 x 10^7^ items/L (>25 mg/L) |  |  |
|  |  |  |  |  | **F_1_** sex ratio | LOEC | >5.2 x 10^7^ items/L (>25 mg/L) |  |  |
|  |  |  |  |  | **F_1_** fecundity (nauplii/ female, 1^st^ brood) | LOEC | ≤2.6 x 10^5^ items/L (≤0.125 mg/L)^c^ |  |  |

| **Test species** | **Test method (duration)** | **Particle type, size** | **Particle concentrations** | | **Endpoint** | **Effect concentration** | **Remark** | **Reference** |
| --- | --- | --- | --- | --- | --- | --- | --- | --- |
|  |  |  | **Numerical conc.** | **Mass conc.** |  |  |  |  |
| **Isopods** | | | | | | | | |
| *Idotea emarginata* | Chronic test (6–7 weeks) with juveniles | Fluorescent microspheres (10 µm) | Approx. 12 items/mg food | – | Survival | No significant effect | – | Hämer et al. [156] |
|  |  | Fluorescent PS fragments (1–100 µm) | 20 Fragments /mg food | – | Growth (length) | No significant effect |  |  |
|  |  | Fluorescent acrylic fibres (20 µm–2.5 mm) | – | 0.3 mg/g food | Duration of the intermoult period | No significant effect |  |  |
| **Molluscs** | | | | | | | | |
| *Mytilus edulis* | 3 h exposure followed by 48 d post-exposure period (in control water) | Fluorescent polystyrene microspheres: (a) 3.0 µm,  (b) 9.6 µm | 4.3 x 10^4^ items/L | n.i. | Feeding rate | No significant effect | All endpoints were evaluated on d 3, 6, 12, 24 and 48 of the post-exposure period | Browne et al. [145] |
|  |  |  |  |  | Haemocyte viability | No significant effect |  |  |
|  |  |  |  |  | Phagocytic activity of the haemocytes | No significant effect |  |  |
|  |  |  |  |  | Capacity of haemocytes to cope with oxidative stress | No significant effect |  |  |
| *M. edulis* | Exposure for 3, 6, 12, 24, 48 and 96 h | High-density polyethylene fluff, 0–80 µm | 2.7–3.6 x 10^7^ items/L (with 8.2 x 10^5^–3.9 x 10^6^ items/L ≤35 µm) | 2.5 g/L | Condition index | No significant effect | Numerical concentration: N. von Moos, pers. comm. | von Moos et al. [152] |
|  |  |  |  |  | Formation of granulocytomas | Significant increase |  |  |
|  |  |  |  |  | Lysosomal membrane stability | Significant decrease |  |  |
|  |  |  |  |  | Neutral lipid content | No significant effect |  |  |
|  |  |  |  |  | Lipofuscin accumulation in digestive tract | No significant effect |  |  |

| **Test species** | **Test method (duration)** | | **Particle type, size** | | **Particle concentrations** | | | **Endpoint** | **Effect concentration** | | **Remark** | | **Reference** |
| --- | --- | --- | --- | --- | --- | --- | --- | --- | --- | --- | --- | --- | --- |
|  |  |  |  |  | **Numerical conc.** | | **Mass conc.** |  |  |  |  |  |  |
| *M. edulis* | 14-d water/sedi­ment test | PS micro­spheres, 10, 30 and 90 µm | | 1.1 x 10^5^ items/L  (10 µm: 5 x 10^4^ items/L, 30 µm: 5 x 10^4^ items/L, 90 µm: 10^4^ items/L) | | n.i. | | Protein content | No significant effect | | – | | Van Cauwenberghe et al. [39] |
|  |  |  |  |  |  |  |  | Carbohydrate content | No significant effect | |  |  |  |
|  |  |  |  |  |  |  |  | Lipid content | No significant effect | |  |  |  |
|  |  |  |  |  |  |  |  | Energy consumption | Significant reduction | |  |  |  |
|  |  |  |  |  |  |  |  | Overall energy budget (cellular energy allocation) | No significant effect | |  |  |  |
| **Fish** | | | | | | | | | | | | | |
| *Pomato-schistus microps* | Acute test with juvenile fish (96 h) | | Red polyethylene microspheres, 1–5 µm | | n.i. | | 18.4 and 184 µg/L | Survival | | No mortality | | – | Oliveira et al. [176] |
|  |  |  |  |  |  |  |  | Acetylcholinesterase activity in head homogenate | | Significant reduction (to approx. 80% of control value) at both concentrations | | Similar reduction in fish co-treated with pyrene |  |
|  |  |  |  |  |  |  |  | Lipid peroxidation | | No significant effect | | – |  |
|  |  |  |  |  |  |  |  | Activity of NADP+-dependent isocitrate dehydrogenase | | No significant effect | | – |  |
|  |  |  |  |  |  |  |  | Activity of glutathione S–transferase | | No significant effect | | – |  |

| **Test species** | **Test method (duration)** | **Particle type, size** | **Particle concentrations** | | | **Endpoint** | **Effect concentration** | | **Remark** | **Reference** |
| --- | --- | --- | --- | --- | --- | --- | --- | --- | --- | --- |
|  |  |  | **Numerical conc.** | **Mass conc.** | |  |  |  |  |  |
| **Studies with marine organisms: water/sediment systems** | | | | |  |  |  |  |  |  |
| **Annelids** |  |  |  | |  |  |  |  |  |  |
| *Arenicola marina* | Chronic sediment/ water test (28 d) | Unplasticised polyvinyl-chloride (uPVC) powder: granules with a mean size of 130 µm and an irregular surface | n.i. | | 5, 10 and 50 g/kg sediment ww (0.5, 1 and 5% w/w, relative to sediment ww) | Feeding rate (number of faecal casts on the sediment surface) | LOEC, weeks 1 and 2 | 50 g/kg sediment ww (5% w/w) | – | Wright et al. [177] |
|  |  |  |  |  |  |  | LOEC, week 3 and 4 | >50 g/kg sediment ww (>5% w/w) |  |  |
|  |  |  |  |  |  | Weight (d 28) | LOEC | >50 g/kg sediment ww (>5% w/w) |  |  |
|  |  |  |  |  |  | Energy reserves (d 28) | LOEC | 10 g/kg sediment ww (1% w/w; approx. 8 x 10^5^ items/kg sediment ww)^d^ |  |  |
|  |  |  |  |  |  | Phagocytic activity of coelomic fluid (d 28) | Significant increase at 5 and 50 g/kd sediment w/w, but not at 10 g/kg sediment ww | |  |  |
| *A. marina* | Exposure for 14 d | PS micro-spheres, 10, 30 and 90 µm | 1.1 x 10^5^ items/kg sediment  (10 µm: 5 x 10^4^ items/kg, 30 µm: 5 x 10^4^ items/kg, 90 µm: 10^4^ items/kg) | | n.i. | Protein content | Significant reduction | | – | Van Cauwenberghe et al. [39] |
|  |  |  |  |  |  | Carbohydrate content | No significant effect | |  |  |
|  |  |  |  |  |  | Lipid content | No significant effect | |  |  |
|  |  |  |  |  |  | Energy consumption | No significant effect | |  |  |
|  |  |  |  |  |  | Overall energy budget (cellular energy allocation) | No significant effect | |  |  |

| **Test species** | **Test method (duration)** | **Particle type, size** | **Particle concentrations** | | **Endpoint** | **Effect concentration** | | **Remark** | **Reference** |
| --- | --- | --- | --- | --- | --- | --- | --- | --- | --- |
|  |  |  | **Numerical conc.** | **Mass conc.** |  |  |  |  |  |
| **Studies with freshwater organisms: water-only systems** | | | |  |  |  |  |  |  |
| **Fish** |  |  |  |  |  |  |  |  |  |
| *Oryzias latipes* | Chronic (2 months) exposure via food | Low density polyethylene pellets ground to <500 µm | n.i. | 10% (w/w) in food | Survival | No effect | | The food containing microplastics had a lower dextrin content (i.e. a lower energy density). All effects were evaluated after month 1 and 2 | Rochman et al. [178, 179] |
|  |  |  |  |  | Glycogen depletion in liver | Strong effect in 46% of the fish | |  |  |
|  |  |  |  |  | Fatty vacuolar degeneration in liver | Slight increase | |  |  |
|  |  |  |  |  | Single cell necrosis in liver | No effect | |  |  |
|  |  |  |  |  | Gonad histology in male and female fish | No effect | |  |  |
|  |  |  |  |  | Expression of *cyp1a* in male and female fish | No significant effect | |  |  |
|  |  |  |  |  | Expression of vitellogenin I in male and female fish | No significant effect | |  |  |
|  |  |  |  |  | Expression of choriogenin H in male fish | No significant effect | |  |  |
|  |  |  |  |  | Expression of choriogenin H in female fish | Significant reduction after 2 months exposure | |  |  |
|  |  |  |  |  | Expression of estrogen receptor α in male and female fish | No significant effect | |  |  |

^a^ Only data for exposure to microplastics only (i.e. no simultaneous exposure to contaminants) are included.

^b^ Several studies only included 1 or 2 test concentrations and, thus, do not allow deriving a LOEC.

^c^ Effect at lowest tested concentration.

^d^ Own approximation based on a particle density of 1.4 g/cm^3^, a mean particle diameter of 130 µm [177] and the simplifying assumption of a spherical form of the particles, and an average wet to dry sediment weight ratio of 1.25 based on [105] and [110].

n.i.: not indicated

**Supplementary references**

S1. Collignon A, Hecq JH, Galgani F, Collard F, Goffart A. Annual variation in neustonic micro- and meso-plastic particles and zooplankton in the bay of Calvi (Mediterranean-Corsica). Mar Pollut Bull. 2014;79:293-298.

S2. Faure F, Saini C, Potter G, Galgani F, de Alencastro LF, Hagmann P. An evaluation of surface micro- and mesoplastic pollution in pelagic ecosystems of the Western Mediterranean Sea. Environ Sci Pollut Res Int. 2015;22:12190-12197.

S3. Griffiths BS, Boag B,Neilson R, Palmer L. The use of colloidal silica to extract nematodes from small samples of soil or sediment. Nematologica 1990;36:465-473.

S4. Day RH, Shaw DG. Patterns in the abundance of pelagic plastic and tar in the north Paciﬁc Ocean, 1976-1985. Mar Pollut. Bull. 1987;18:311-316.

S5. Algalita Marine Research Foundation 2002–2011. Mapping plastic pollution: GIS Maps of plastic density in the North Paciﬁc subtropical gyre (NPSG). http://www.algalita.org/research/Maps_Home.html.

S6. Doyle MJ, Watson W, Bowlin NM, Sheavly SB. Plastic particles in coastal pelagic ecosystems of the Northeast Paciﬁc Ocean. Mar Environ Res 2011;71:41-52.

All other references are included in the reference list for the main text.
